# Contextual effects of mesenchymal stem cell injections for knee osteoarthritis: systematic review and meta-analysis of randomized controlled trials

**DOI:** 10.3389/fmed.2025.1636181

**Published:** 2025-09-17

**Authors:** Fengsheng Yin, Houdong Wu, Dan Tong, Gan Luo, Zhen Deng, Qinyi Yan, Yu Zhang

**Affiliations:** ^1^Department of Orthopedics, Chengdu Integrated TCM & Western Medicine Hospital, Chengdu University of Traditional Chinese Medicine, Chengdu, China; ^2^School of Acupuncture and Massage Therapy, Chengdu University of Traditional Chinese Medicine, Chengdu, China; ^3^School of Clinical Medicine, Chengdu University of Traditional Chinese Medicine, Chengdu, China

**Keywords:** mesenchymal stem cells, knee osteoarthritis, contextual effects, meta-analysis, intra-articular injection

## Abstract

**Purpose:**

To quantify the proportion of the overall clinical improvement produced by intra-articular mesenchymal stem cell (MSC) injections for knee osteoarthritis (KOA) that is attributable to contextual (placebo-related) effects.

**Methods:**

This PRISMA-compliant systematic review and meta-analysis (PROSPERO CRD420251026818) searched five databases (CENTRAL, Embase, MEDLINE, Web of Science and Scopus) to 24 March 2025. Randomized controlled trials enrolling adults with KOA that compared MSC injections with inert placebo were included. Primary outcome was change in pain intensity (VAS or WOMAC-pain); physical function was analysed secondarily. Two reviewers independently extracted data and assessed risk of bias. The proportion of the treatment effect attributable to contextual factors (PCE) was calculated as described by Tsutsumi et al. Pain and function outcomes at 6 and 12 months were pooled with inverse-variance random-effects meta-analysis, and evidence certainty was appraised using GRADE.

**Results:**

Eight RCTs (467 patients) met the inclusion criteria. At 6 months, contextual factors accounted for approximately 63% of pain reduction and 61% of functional improvement, with low heterogeneity (*I*^2^ ≤ 8%). At 12 months, contextual factors explained ~50% of pain relief and ~66% of functional gains, again with very low heterogeneity (*I*^2^ = 0%). Certainty of evidence was rated low for both time-points (GRADE).

**Conclusion:**

Based on low-certainty evidence, this meta-analysis suggests that in knee osteoarthritis the majority of symptomatic improvement following intra-articular MSC injections is attributable to contextual (placebo) effects, whereas the MSCs themselves confer only a modest incremental benefit.

**Systematic review registration:**

CRD4-2025-1636181, https://www.crd.york.ac.uk/PROSPERO/view/CRD420251026818.

## 1 Introduction

Knee osteoarthritis (KOA) is a progressive degenerative joint disease that causes chronic pain, stiffness, and functional limitations, and is a leading cause of disability and reduced quality of life in older adults (1). Its prevalence increases with age, affecting approximately 10% of men and 13% of women over 60 years old, which equates to over 250 million people worldwide (2). The burden of KOA is expected to continue rising given increasing life expectancy (3) and the growing obesity epidemic (4). Current treatments—including weight loss, physical therapy, exercise, analgesic medications (e.g., NSAIDs), and intra-articular corticosteroid or hyaluronic acid injections—provide symptomatic relief but do not regenerate cartilage or halt disease progression (2). In advanced cases, many patients ultimately progress to total knee arthroplasty (4). In the past decade, regenerative medicine approaches using mesenchymal stem cells (MSCs) derived from bone marrow, adipose tissue, or umbilical cord have emerged as a potential therapeutic strategy for treating KOA. MSCs can modulate the inflammatory environment and stimulate cartilage repair, owing to their immunomodulatory and chondrogenic properties (5, 6), theoretically enabling them not only to reduce the inflammation that drives pain but also to actively contribute to the regeneration of damaged cartilage tissue. Early clinical studies indicate that MSC therapy may alleviate pain and improve function, and importantly, these cell-based therapies have demonstrated a favorable safety profile in clinical trials (7).

Despite this potential, the evidence supporting the efficacy of MSC injections from randomized controlled trials (RCTs) remains limited (8, 9). Several systematic reviews and meta-analyses evaluating intra-articular MSC therapy for KOA have consistently found only modest improvements in pain and function compared to placebo or other active controls (9–11). For example, a recent Cochrane review reported that MSC treatment provided just a 1.2-point greater reduction in pain (on a 0–10 scale) and about a 14.2-point greater improvement in WOMAC functional score (0–100 scale) compared to placebo (11). These small between-group differences contrast with the often larger reported improvements observed in patients receiving MSCs in routine practice or open-label studies, where patients often report considerable pain relief and functional gains (12). This discrepancy suggests that a significant portion of the symptomatic benefit from MSC therapy may be driven by placebo and other contextual effects rather than the cell product alone (13). In other words, the traditional focus on between-group “specific” effects may underestimate the total improvement patients experience, since non-specific factors contribute markedly to outcomes—a situation termed the “efficacy paradox” (14).

Placebo and other contextual factors can indeed account for a large proportion of the improvements seen in KOA treatment outcomes (15). Various non-specific elements—such as patients’ expectations and beliefs (16), a supportive patient–provider relationship, the perceived novelty and credibility of a regenerative therapy (17), and the act of receiving an invasive injection (18)—may all amplify clinical outcomes beyond the specific biological effect of the cells (16). Additionally, natural fluctuations in symptoms and regression to the mean also contribute to the overall response (19). Collectively, these factors alongside the treatment’s direct effects constitute the total effect experienced by the patient. Recent research has attempted to quantify the magnitude of such contextual influences. Notably, Zou et al. analyzed clinical trials of osteoarthritis treatments and found that approximately 75% of the overall pain relief observed was attributable to contextual effects (20). However, no prior review has specifically examined how much of the benefit from MSC therapy in KOA is due to contextual mechanisms versus the cells’ specific effects. Therefore, this study aimed to evaluate MSC injection outcomes in KOA through a systematic review and meta-analysis, with an emphasis on disentangling and quantifying the contextual component of the treatment response. In particular, the focus was placed on pain and physical function improvements, as these are core outcome domains recommended for assessment in OA trials (21).

## 2 Method

### 2.1 Study design and registration

This review adhered to the Preferred Reporting Items for Systematic Reviews and Meta-Analyses (PRISMA) guidelines for healthcare intervention reviews (22). The comprehensive PRISMA checklist is provided in Supplementary File 1. The protocol was prospectively registered in PROSPERO (registration no. CRD420251026818; available from https://www.crd.york.ac.uk/PROSPERO/view/CRD420251026818).

### 2.2 Search strategy

A medical librarian (Yin) designed and executed comprehensive, language-unrestricted search strategies across five electronic databases—Cochrane CENTRAL via Wiley, EMBASE via Elsevier, MEDLINE via PubMed, Web of Science, and Scopus—from their inception through 24 March 2025. Searches combined controlled vocabulary (e.g., MeSH) and free-text terms for “mesenchymal stem cells” and “knee osteoarthritis.” The full search syntaxes for each database are detailed in Supplementary File 2.

### 2.3 Eligibility criteria and study selection

Two reviewers (Yin and Wu) independently screened titles, abstracts, and keywords against predefined eligibility criteria; disagreements were resolved by discussion or, if needed, consultation with a third reviewer.

Randomized controlled trials enrolling adults (≥18 years) with clinically and/or radiologically confirmed knee osteoarthritis that compared intra-articular injections of mesenchymal stem cells—from any source (e.g., bone marrow, adipose tissue, umbilical cord, placenta)—against an inert placebo (e.g., physiological saline or electrolyte solution) were included.

Studies were excluded if they (1) involved prior knee arthroplasty or other major surgery on the index knee; (2) used non-MSC cellular therapies (e.g., micro-fragmented adipose tissue, chondrocyte implants, bone marrow aspirate concentrate, peripheral blood stem cells); (3) were animal or *in vitro* studies; (4) were non-primary research articles (e.g., reviews, editorials, letters, conference abstracts without sufficient data); (5) represented previous systematic reviews or meta-analyses; or (6) lacked full-text availability.

### 2.4 Data extraction and management

Data were independently extracted by two authors (Yin and Tong) using a standardized form, and any discrepancies were resolved by consensus. Extracted variables included publication details (first author, year, country); participant characteristics (sample size, mean age, mean body mass index, Kellgren–Lawrence grade); details of the mesenchymal stem cell (MSC) intervention (cell source and dose) and the comparator (placebo type and dosage); pain and functional outcome scores at baseline and follow-up; and follow-up duration.

Mean changes from baseline and corresponding standard deviations (SDs) were recorded when reported or calculated from baseline and follow-up data. To address missing data, study authors were contacted and, when data remained unavailable, missing means or SDs were derived from other reported summary statistics (23), extracting graphical data via digitization software (23). For trials with multiple dosage arms compared against a single control, each arm was treated as a separate comparison (23). When only absolute baseline and follow-up values were provided, mean change scores were calculated and within-group SDs estimated using reported correlations; if correlations were not reported, the coefficient (*r*) was imputed based on analogous studies (23).

### 2.5 Risk of bias assessment

The risk of bias was independently assessed by two reviewers (Yin and Tong) using the Cochrane Risk of Bias tool in accordance with the Cochrane Handbook (23); disagreements were adjudicated by a third reviewer (Wu).

### 2.6 Statistical analysis

In the meta-analysis, the proportion of the MSC treatment effect attributable to contextual (placebo-related) factors—the proportion attributable to contextual effects (PCE)—was calculated following the method of Tsutsumi et al. (24). The PCE represents the fraction of the observed MSC treatment response that can be explained by non-specific (placebo) influences (24). For each included study and outcome (pain and functional outcomes), effect sizes were calculated as the within-group mean change from baseline to follow-up divided by the within-group standard deviation (25). The MSC treatment effect size was defined in the MSC arm, and the contextual effect size in the placebo arm. Outcome measurements at 6–12 months post-intervention were used for all effect size calculations. The PCE for each outcome was then derived as the ratio of the contextual effect size to the overall MSC effect size (24). Statistical heterogeneity among studies was assessed using the *τ*^2^ and *I*^2^ statistics (26), with Cochran’s *Q* (*χ*^2^) test *p* ≤ 0.10 indicating significant heterogeneity (23). Because MSC protocols varied across trials, all pooled analyses employed inverse-variance–weighted random-effects models to accommodate between-study variability (23). When at least 10 studies were available for a meta-analysis, publication bias was evaluated by visual inspection of funnel plots and Egger’s regression test (23, 27). All statistical analyses were performed in R (version 4.3.3).

### 2.7 Certainty of evidence assessment

Evidence certainty for each pooled outcome was appraised with the GRADE approach in GRADEpro GDT (23, 28), beginning at “high” because all included studies were randomized controlled trials. Certainty was then downgraded by one “–1” or two “–2” levels across five domains: (1) risk of bias: certainty was downgraded by one level (−1) when 50% or more of the included studies were rated at high risk of bias according to RoB 2.0; (2) inconsistency:–1 when *I*^2^ > 50% or the 95% prediction interval crossed the null; (3) indirectness:–1 when populations, interventions, comparators, or outcome measures diverged materially from the review question; (4) imprecision, downgraded (−1) if the 95% CI for the pooled ROM included 1.0 or if total sample size was <400 participants. (28, 29); and (5) publication bias, one level if the funnel plot indicates that publication bias could be present. In case funnel plots could not be inspected (less than 10 studies) this domain was considered low risk. Final ratings (“high,” “moderate,” “low,” or “very low”).

## 3 Results

### 3.1 Study selection

**Figure 1:**
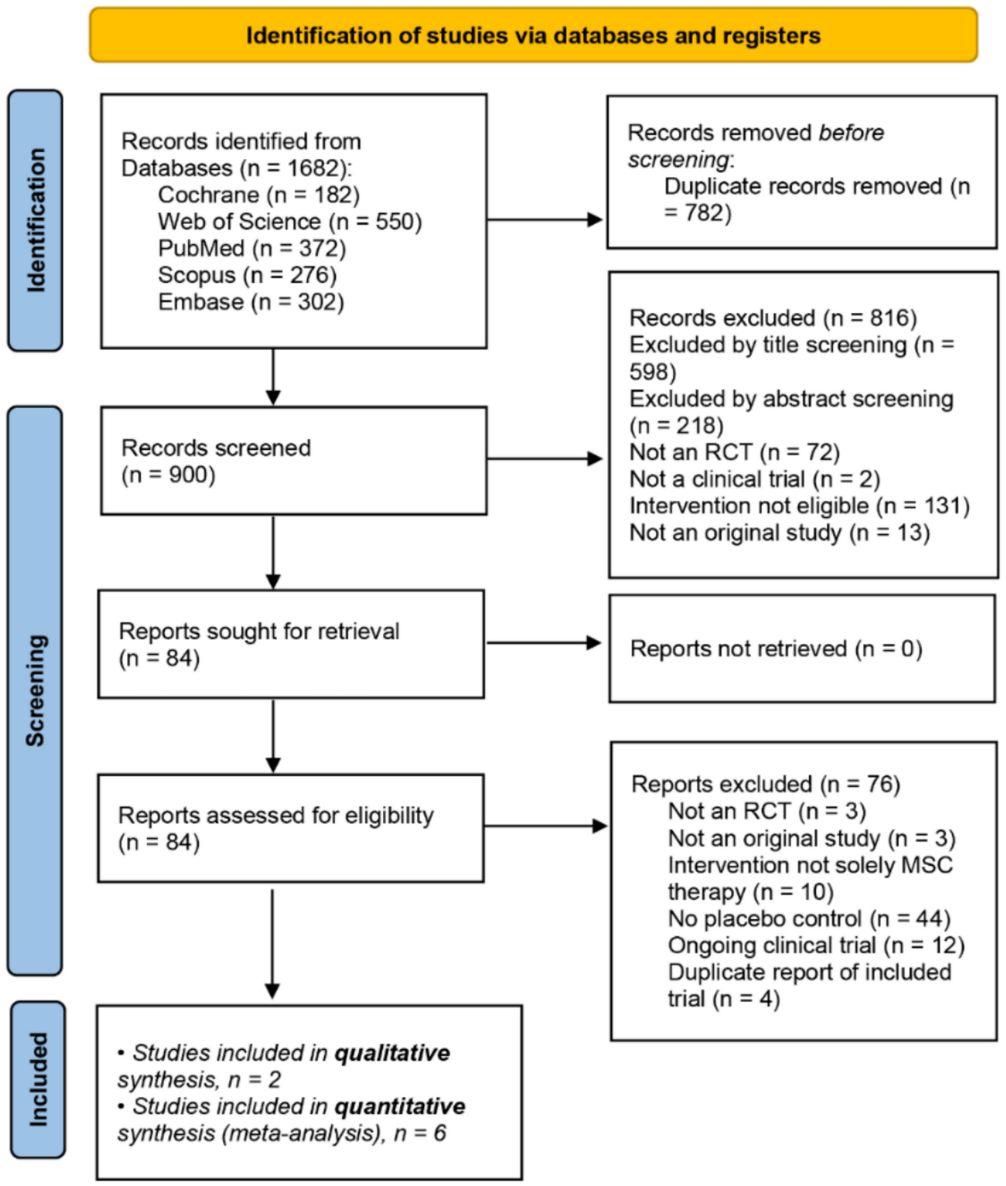
PRISMA flow diagram of the study selection process.

A total of 1,682 records were identified through database searches. After removing 782 duplicate records, 900 unique records remained. Title and abstract screening excluded 816 records, leaving 84 articles for full-text assessment. Ultimately, eight randomized controlled trials—including 477 patients—met the eligibility criteria and were included in the systematic review (30–37). The PRISMA flow diagram in Figure 1 summarizes the study selection process (22). A detailed list of excluded studies and their reasons for exclusion can be found in Supplementary File 3.

### 3.2 Study characteristics

Eight randomized trials enrolled 467 participants with Kellgren–Lawrence grade II–IV KOA (per-trial *n* = 20–261; mean age 47.6–67.2 years; mean BMI 25.0–30.9 kg m^−2^) (30–37). Each study delivered a single intra-articular MSC injection: five used adipose-derived MSCs (31, 33, 34, 36, 37), two used bone-marrow-derived MSCs (30, 35), and one used placenta-derived MSCs (32). Administered doses ranged from 3.9 × 10^6^ to 100 × 10^6^ cells. Control arms received inert placebo—saline (30, 32, 33, 35–37), Plasmalyte (31), 2% human serum albumin (30, 33), or Cryoprotectant-containing culture medium (34). Follow-up intervals ranged from 1 week to 12 months, with outcomes assessed by VAS, WOMAC pain and function subscales, KOOS-ADL, and other daily-function measures.

Six trials reported numerical results suitable for quantitative pooling (30, 31, 33–35, 37); the remaining two, which provided only graphical trends, were synthesized qualitatively (32, 36). In one study, mean change scores were calculated from published baseline and follow-up values to facilitate cross-study comparability (37). A concise overview of trial designs, intervention details, and participant characteristics is presented in Table 1.

**Table 1:** Summary of characteristics of included randomized controlled trials.

Authors (year)	Country	Participants Age K-L grade BMI	Cell dose	Cell type, source	Placebo intervention	Follow up	Measurement instruments
Freitag et al. (2024) (31)	Australia	*N* = 40 Mean age = 47.64 K-L grade = 2–3 Mean BMI = 26.99	E1 = 10 × 10^6^ cells (*N* = 8) E1 = 20 × 10^6^ cells (*N* = 8) E1 = 50 × 10^6^ cells (*N* = 8) E1 = 100 × 10^6^ cells (*N* = 8)	Allogeneic ADMSCs	5 mL plasma-lyte 148 IV-infusion (*N* = 8)	1 m, 3 m, 6 m, 9 m, 12 m	NPRS, KOOS ADL
Emadedin et al. (30)	Iran	*N* = 47 Mean age = 53.37 K-L grade = 2–4 Mean BMI = 30.93	40 × 10^6^ cells (*N* = 19)	Autologous BM-MSCs	5 mL saline + 2% HSA (*N* = 24)	1 wk., 3 m, 6 m	VAS, WOMAC function
Soltani et al. (2019) (32)	Iran	*N* = 20 Mean age = 56.65 K-L grade = 2–4 Mean BMI = 29.25	50–60 × 10^6^ cells (*N* = 10)	Allogeneic PLMSCs	10 mL normal saline (*N* = 10)	2 wk., 8 wk., 24 wk	VAS, KOOS ADL
Sadri et al. (2023) (37)	Iran	*N* = 40 Mean age = 54.48 K-L grade = 2–3 Mean BMI = 28.75	100 × 10^6^ cells (*N* = 20)	Allogeneic ADMSCs	5 mL normal saline (*N* = 20)	3 m, 6 m, 12 m	VAS, KOOS Daily function
Kim et al. (2023) (33)	South Korea	*N* = 261 Mean age = 63.75 K-L grade = 3 Mean BMI = 26.10	100 × 10^6^ cells (*N* = 125)	Autologous ADMSCs	2.1 mL normal Saline + 0.9 mL autologous serum (*N* = 127)	1 m, 3 m, 6 m	100-mm VAS on pain, WOMAC Function subscore
Kuah et al. (2018) (34)	Australia	*N* = 21 Mean age = 53.32 K-L grade = 1–3 Mean BMI = 26.9	PRG 3.9 M = 3.9 × 10^6^ cells (*N* = 8) PRG 6.7 M = 6.7 × 10^6^ cells (*N* = 8)	Allogeneic ADMSCs	2 mL placebo (cell culture media + cryopreservative) (*N* = 8)	Day 7, Day 28, 3 m, 6 m, 9 m, 12 m	VAS pain (0–100 mm), WOMAC function (0–68)
Lee et al. (2025) (35)	South Korea	*N* = 24 Mean age = 67.2 K-L grade = 2–4 Mean BMI = 25.00	100 × 10^6^ cells (*N* = 11)	Allogeneic BM-MSCs	2 mL normal saline (*N* = 12)	3 m, 6 m, 9 m, 12 m	VAS pain (0–100 mm), WOMAC function (0–68)
Lee et al. (2019) (36)	South Korea	*N* = 24 Mean age = 62.7 K-L grade = 2–4 Mean BMI = 25.35	100 × 10^6^ cells (*N* = 12)	Autologous ADMSCs	3 mL saline (*N* = 12)	1 m, 3 m, 6 m	VAS, WOMAC function

ADL, activities of daily living; ADMSCs, adipose-derived mesenchymal stem cells; Allogeneic, from a genetically non-identical donor of the same species; Autologous, from the patient’s own body; BM-MSCs, bone marrow-derived mesenchymal stem cells; BMI, body mass index; HAS, human serum albumin; K-L, Kellgren–Lawrence; KOOS, knee injury and osteoarthritis outcome score; M, million (106); m, month (s); N, number of participants; NPRS, numeric pain rating scale; PLMSCs, placenta-derived mesenchymal stem cells; Plasma-Lyte 148 IV-Infusion, An electrolyte solution with a composition similar to human plasma; VAS, visual Analogue Scale; wk, week (s); WOMAC, Western Ontario and McMaster Universities Osteoarthritis Index.

### 3.3 Risk of bias

**Figure 2:**
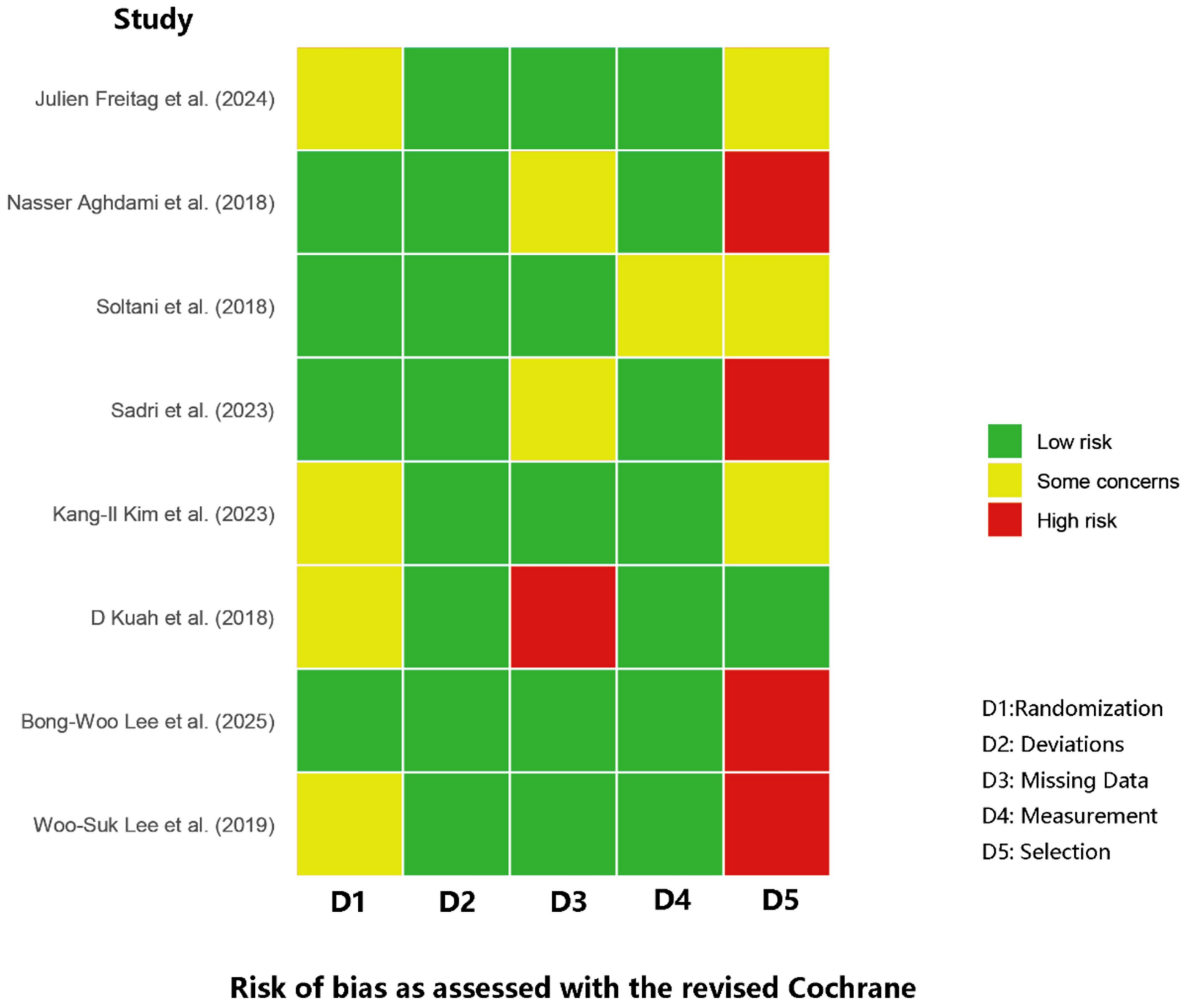
Summary of risk of bias assessment for included studies.

Risk of bias was assessed across all eight RCTs by two independent reviewers, yielding substantial agreement (*κ* = 0.77). Four trials showed concerns about the randomization process—chiefly inadequate allocation concealment (31, 33, 34, 36). A single trial was judged at high risk for missing outcome bias due to an influential dropout event: a patient in the treatment arm withdrew to undergo knee replacement surgery, an outcome directly related to treatment failure that was not adequately accounted for in the analysis (34). Measurement bias was generally low, yet selective-reporting bias was a concern: four studies were rated high risk due to various outcome reporting biases, including inconsistencies between registered and published primary endpoints, an emphasis on positive secondary outcomes, and internal contradictions regarding the primary outcome’s definition (30, 35–37). Overall study quality therefore ranged from “some concerns” to “high risk,” with randomization and selective reporting emerging as the predominant sources of bias. Figure 2 offers a visual synthesis of these findings.

### 3.4 Meta-analysis

Six of the eight eligible randomized controlled trials provided extractable numerical data for quantitative pooling (30, 31, 33–35, 37). The remaining two pilot studies [Soltani et al. (32); Lee et al. (36)] reported outcomes only in graphical form or without sufficient measures of variance and were therefore synthesized qualitatively. Qualitatively, the study by Lee et al. (36) reported that a single autologous MSC injection led to significant improvements in pain and function at 6 months compared to the saline control. In contrast, the pilot study by Soltani et al. (32) found that allogeneic MSCs offered only transient clinical improvements, with no significant difference in VAS pain reduction compared to placebo at the 24-week endpoint.

### 3.5 Six-month outcomes

**Figure 3:**
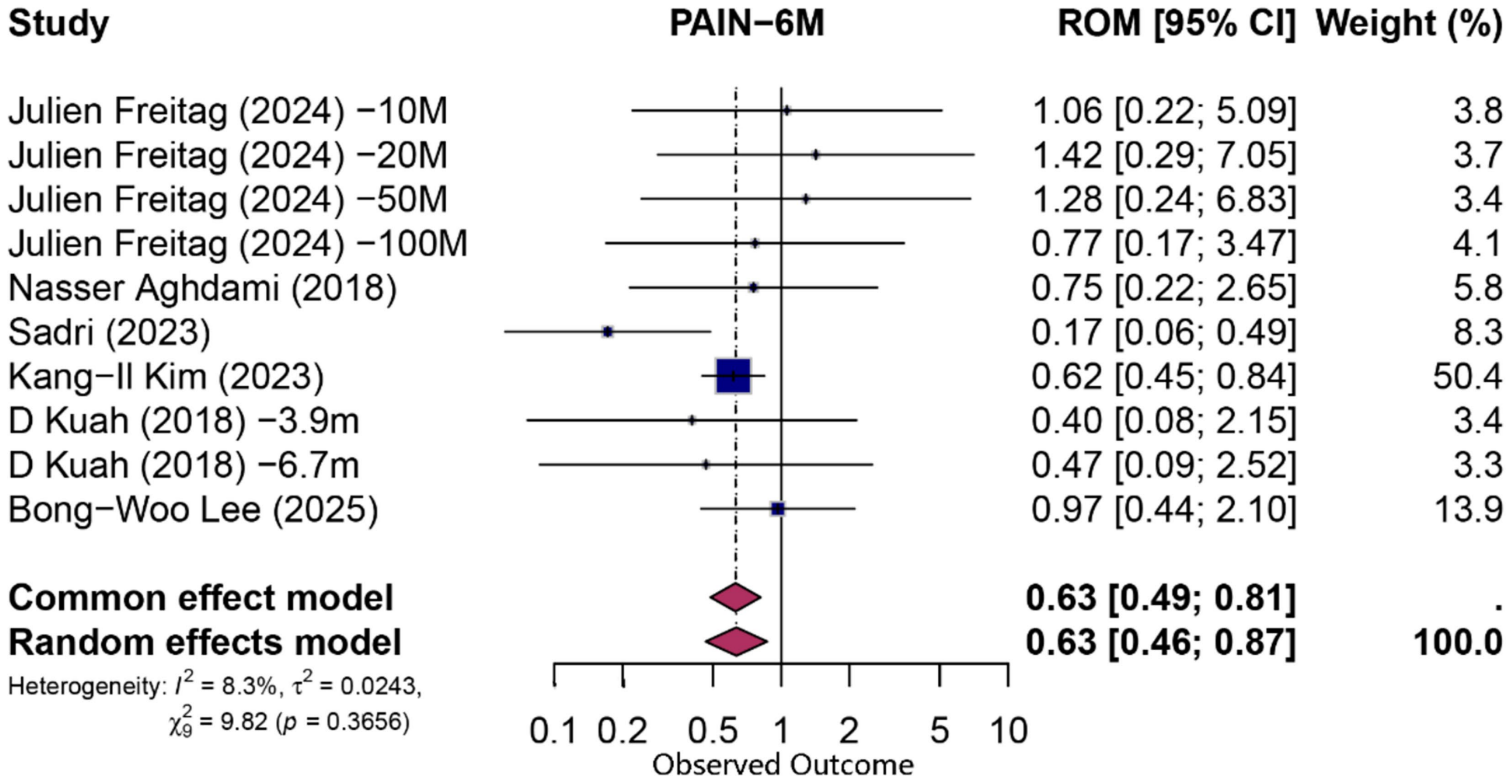
Forest plot of 6-month proportion attributable to contextual effects for pain.

**Figure 4:**
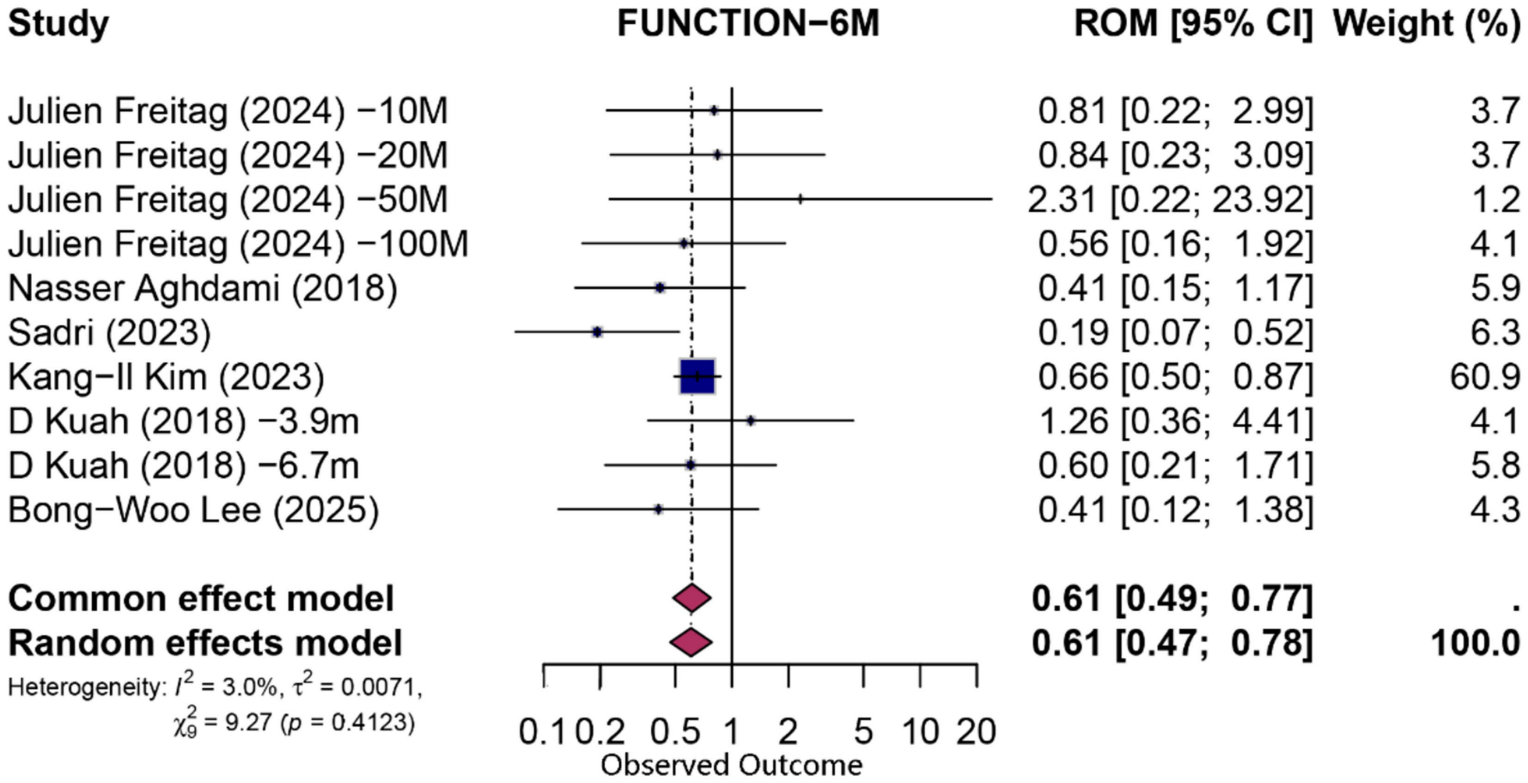
Forest plot of 6-month proportion attributable to contextual effects for function.

At 6 months, pooling data from 10 comparisons (derived from six studies) assessing functional improvement yielded a statistically significant overall proportion attributable to contextual effects (PCE) of 0.61 (95% CI 0.47–0.78; *p* = 0.0001; *I*^2^ = 3%, random-effects model), indicating that approximately 61% of the observed functional gains could be attributed to non-specific (contextual) factors. Similarly, pain reduction at 6 months (*n* = 10 comparisons from six studies) demonstrated a significant contextual contribution (PCE = 0.63; 95% CI 0.46–0.87; *p* = 0.004; *I*^2^ = 8.3%). Forest plots for 6-month functional and pain outcomes are shown in Figures 3, 4, respectively.

### 3.6 Twelve-month outcomes

**Figure 5:**
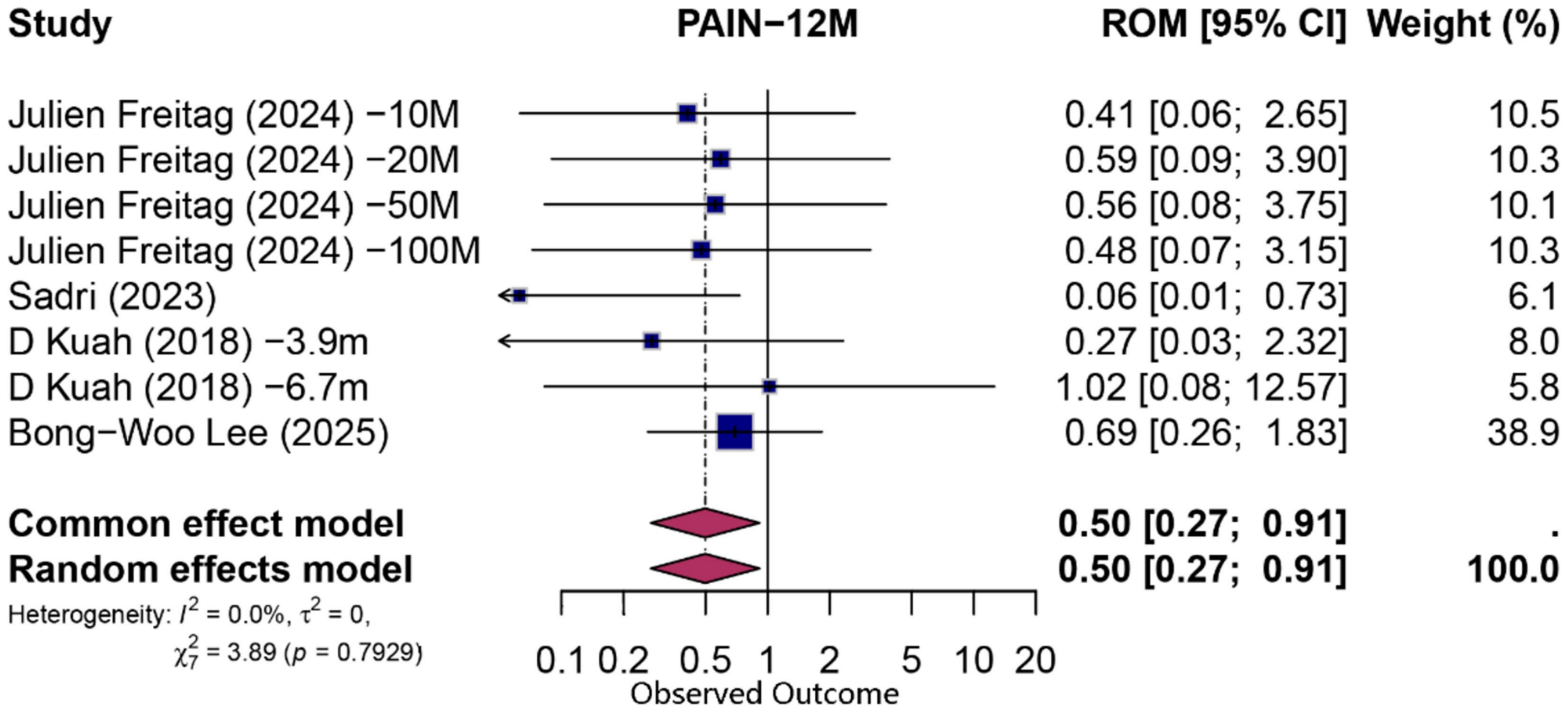
Forest plot of 12-month proportion attributable to contextual effects for pain.

**Figure 6:**
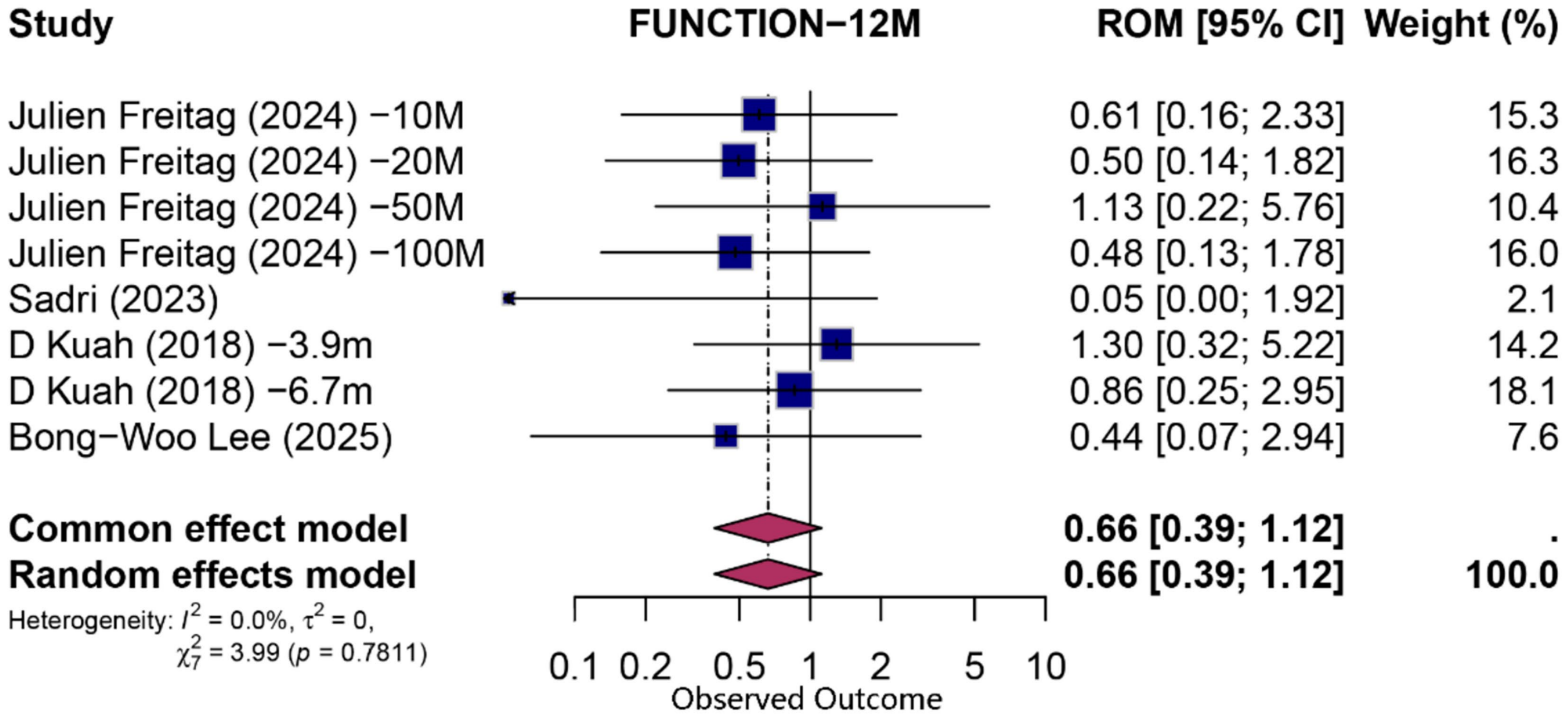
Forest plot of 12-month proportion attributable to contextual effects for function.

At 12 months, eight comparisons (derived from four studies) contributed to both functional and pain analyses. For function, the pooled PCE was 0.66 (95% CI 0.39–1.12; *p* = 0.124; *I*^2^ = 0%), indicating no statistically significant specific effect beyond contextual influences. Pain outcomes exhibited a modest contextual contribution (PCE = 0.50; 95% CI 0.27–0.91; *p* = 0.024; *I*^2^ = 0%). Detailed forest plots for 12-month outcomes are presented in Figures 5, 6.

### 3.7 Heterogeneity

Overall heterogeneity was low to moderate, with no analysis showing statistically significant between-study heterogeneity (all Cochran’s *Q p* > 0.05). However, prediction intervals were wide—for example, the 95% prediction interval for 6-month functional outcomes ranged from 0.43 to 0.86—reflecting variability among individual trials.

### 3.8 Sensitivity analysis

A leave-one-out sensitivity analysis was conducted for all pooled outcomes to assess the stability of the findings, with detailed results presented in Supplementary File 4.

The analysis confirmed that the statistical significance of the 6-month pain outcome was highly dependent on the inclusion of the Kim et al. (33) trial, which was the most influential study. Omitting this single trial produced a pooled Proportion Attributable to Contextual Effects (PCE) of 0.66, with a 95% confidence interval that reached the null value (95% CI 0.43–1.00). In contrast, the primary analysis yielded a PCE of 0.63 (95% CI 0.46–0.87). This confirms that the contextual effect on pain relief at 6 months is sensitive to this single high-weight study.

For functional improvement at 6 months, the finding was robust. Although omitting the Kim et al. (33) trial yielded a slightly lower pooled PCE of 0.54 (95% CI 0.36–0.80), the effect remained statistically significant. The removal of any other study did not substantially alter the primary finding, underscoring the stability of the contextual contribution to functional gains at 6 months.

The sensitivity analyses for the 12-month outcomes indicated that both the significant finding for pain relief and the non-significant finding for functional improvement were robust and not unduly influenced by any single study.

### 3.9 Publication bias

A formal assessment of publication bias was precluded by the limited number of studies included in each meta-analysis. With a maximum of six studies per outcome, statistical tests for funnel plot asymmetry (e.g., Egger’s test) have insufficient power to reliably distinguish true heterogeneity from publication bias. Therefore, in accordance with Cochrane guidelines, these analyses were not performed (23).

### 3.10 Assessment of the body of evidence (GRADE)

The certainty of the evidence for all pooled outcomes at both 6 and 12 months was judged to be low using the Grading of Recommendations Assessment, Development and Evaluation (GRADE) framework.

## 4 Discussion

### 4.1 Main finding

This systematic review of eight randomized trials (*n* = 467) demonstrates that contextual factors are the principal driver of symptomatic improvement following intra-articular MSC injections for knee osteoarthritis. We estimated that contextual influences accounted for approximately 60–63% of the observed pain reduction and functional gain at 6 months, with comparable magnitudes noted at 12 months (≈50–66%). This finding is congruent with evidence that inert intra-articular injections alone can confer statistically and clinically significant pain relief lasting up to 6 months (38). Moreover, it aligns with broader meta-analytic data from osteoarthritis trials, which estimated that ≈75% of overall pain reduction is attributable to contextual factors, and that injection-based placebos elicit stronger responses than oral placebos (“needle is better than pill”) (20, 39). Collectively, these converging findings suggest that the therapeutic ritual surrounding the intervention, rather than the cellular product alone, is responsible for the majority of the clinical benefit.

### 4.2 The MSC ‘efficacy paradox’ and its contextual mechanisms

These observations help explain the “efficacy paradox” of MSC therapy: patients and clinicians often report dramatic symptom improvements after cell injections, yet controlled trials show only a modest additional benefit of MSCs over placebo (8). The results provide a quantitative explanation for these small between-group differences by demonstrating the large magnitude of the non-specific effects. By quantifying PCE, this analysis reconciles this paradox: MSC injections produce meaningful gains in pain and function, but only a small extra fraction (roughly one-third) is attributable to the cells themselves. The remaining benefit derives from a robust therapeutic context, likely driven by factors we previously introduced, such as heightened patient expectations for a novel ‘stem cell’ therapy, the potent placebo effect of an invasive injection ritual, and the natural regression to the mean from peak pain levels at trial enrollment (40, 41). Recognition of this aspect helps align patient experiences with trial results: patients truly feel better, but trials show that most of this improvement is context-driven.

### 4.3 Intervention heterogeneity

Substantial heterogeneity in MSC interventions across trials was observed, which could influence both specific and contextual outcomes (42). The studies differed in MSC source (adipose, bone marrow, or perinatal tissue), cell dose and processing, and other procedural details (such as use of adjuncts or rehabilitation protocols). It is biologically plausible that these factors affect the magnitude of the true treatment effect (43, 44). However, formal subgroup analyses by cell type, dose, or manufacturing method were not performed due to the limited number of trials. Instead, random-effects meta-analytic models were used to account for between-study variability, yielding wide confidence and prediction intervals that reflect uncertainty in any specific setting. Importantly, the PCE estimates remained high across studies (>0.50), implying that even if some MSC products have slightly greater specific efficacy than others, contextual factors dominated the overall response in all subgroups.

### 4.4 Certainty of evidence and risk-of-bias profile

The confidence we place in these findings is tempered by methodological limitations of the primary studies (45). Many included RCTs were small, early-phase or pilot trials with potential risk-of-bias concerns. Blinding is a particular challenge in cell-therapy trials (46): although most studies attempted sham injections, subtle cues (such as differences in injectate appearance or transient local reactions) could inadvertently unblind patients or assessors. Incomplete outcome data, selective reporting, and other trial-level issues were also common (23). Therefore, these considerations underscore the need for caution in interpreting the results.

These limitations collectively downgraded the certainty of evidence to low for both the 6- and 12-month time points. Under GRADE, risk-of-bias concerns (small, early-phase trials and imperfect blinding), imprecision (wide prediction intervals centred on modest specific effects) and suspected publication bias each triggered one level of downgrading. Formal funnel-plot or Egger testing was not feasible because fewer than 10 studies were available; nevertheless, our search of trial registries identified several completed but unpublished MSC RCTs, suggesting that negative or null findings may be under-represented.

### 4.5 Limitations

Notwithstanding these caveats, a key strength of this review is its explicit quantification of the contextual component via the PCE ratio. We assumed that the contextual effect equated to the change observed in the placebo arm, implying that all improvements in this group resulted exclusively from placebo mechanisms (e.g., patient expectations, conditioning) or the natural progression of the disease. However, additional factors—such as regression to the mean, mechanical influences of injection (e.g., joint lavage effects)—may also have contributed to observed improvements. Therefore, our estimates of the Proportion Attributable to Contextual Effects (PCE) represent the aggregate impact of all contextual factors and may slightly overestimate the pure psychological placebo effect (47). Several trials did not report essential summary statistics, such as standard deviations for change scores, necessitating extraction of data from published graphical presentations (31, 34) and the imputation of missing correlation coefficients (37) for variance calculations. We employed established methods throughout these processes to minimize potential bias. The limited number of included trials precluded predefined subgroup analyses based on MSC source, dosage, or specific patient characteristics. Additionally, in certain trials, multiple MSC dosage groups shared a single placebo control group (31, 34). While we adhered to standard practices by proportionately dividing shared control groups across comparisons, this approach reduced the effective sample size per comparison and could introduce dependencies or artificially narrow the precision of pooled estimates. Finally, our analysis was restricted to outcomes measured up to 1 year post-intervention due to the lack of longer-term randomized controlled trial (RCT) data. Consequently, our PCE estimates reflect only short- to mid-term responses and may not accurately predict longer-term therapeutic trajectories.

### 4.6 Clinical implications

This review highlights the utility of the PCE framework in clinical trials, supplementing traditional meta-analytic measures like between-group mean differences. Systematic reviews typically focus on mean differences to establish specific efficacy (crucial for regulatory decisions), but focusing only on these can obscure the full clinical picture (20). By analyzing within-group improvements in both the MSC and placebo arms, insight is gained into how much patients improve overall and how that improvement is partitioned between contextual and specific effects.

Presenting PCE alongside standard effect sizes enables a more nuanced and clinically meaningful interpretation. Furthermore, understanding the relative contributions of specific and contextual effects can significantly inform patient communication. It allows for a more transparent discussion about treatment expectations, potentially shifting the focus towards realistic goals for the cellular therapy itself while highlighting the importance of harnessing positive contextual factors and adhering to adjunctive strategies (such as rehabilitation and lifestyle modifications) to maximize overall outcomes (48). These clinical implications directly inform priorities for future research: conducting long-term RCTs with rigorous reporting to assess the durability of these effects, while simultaneously investigating how to enhance the specific efficacy of MSCs and ethically maximize contextual gains.

## 5 Conclusion

This systematic review and meta-analysis indicates that, when intra-articular mesenchymal stem-cell therapy is used for knee osteoarthritis, a substantial portion of the observed symptomatic improvement is attributable to contextual influences. This finding does not necessarily negate the biological potential of MSCs, but rather reveals a critical insight: in the current clinical application for KOA, the specific effects derived from their immunomodulatory and chondrogenic properties are significantly amplified by a powerful therapeutic context. Therefore, while the biological rationale for MSCs remains a compelling area for basic science research, the path to improving patient outcomes may lie in understanding and ethically leveraging both the cellular action and these profound contextual effects. Our estimate, however, rests on low-certainty evidence, underscoring the need for larger, more robust trials to fully dissect these intertwined components.

## Data Availability

The original contributions presented in the study are included in the article/Supplementary material, further inquiries can be directed to the corresponding author.
