# Supplementary material for: Contextual effects of mesenchymal stem cell injections for knee osteoarthritis: systematic review and meta-analysis of randomized controlled trials
Source: Front Med (Lausanne). 2025 Sep 17;12:1636181. doi: 10.3389/fmed.2025.1636181 (PMC12487426; doi:10.3389/fmed.2025.1636181)
Supplement: Supplementary file 2 [file Table_2.docx]

**S1 Table. Search strategies.**

# Source: Pubmed

**Starting date: 2005**

**Searched on:** March 24, 2025

**Results:** 372

| **Search** | **Query** | **Results** |
| --- | --- | --- |
| #1 | (clinical[Title/Abstract] AND trial[Title/Abstract]) OR clinical trials as topic[MeSH Terms] OR clinical trial[Publication Type] OR random*[Title/Abstract] OR random allocation[MeSH Terms] OR therapeutic use[MeSH Subheading] OR randomized controlled trial[Publication Type] OR randomized[Title/Abstract] OR placebo[Title/Abstract] | 6766018 |
| #2 | "Mesenchymal Stem Cells"[Mesh] | 56390 |
| #3 | (Mesenchymal Stem Cells[Title/Abstract]) OR (Stem Cell, Mesenchymal[Title/Abstract]) OR (Stem Cells, Mesenchymal[Title/Abstract]) OR (Mesenchymal Stem Cell[Title/Abstract]) OR (Bone Marrow Mesenchymal Stem Cells[Title/Abstract]) OR (Bone Marrow Mesenchymal Stem Cell[Title/Abstract]) OR (Bone Marrow Stromal Cells[Title/Abstract]) OR (Bone Marrow Stromal Cell[Title/Abstract]) OR (Bone Marrow Stromal Cells, Multipotent[Title/Abstract]) OR (Bone Marrow Stromal Stem Cells[Title/Abstract]) OR (Multipotent Bone Marrow Stromal Cell[Title/Abstract]) OR (Multipotent Bone Marrow Stromal Cells[Title/Abstract]) OR (Adipose-Derived Mesenchymal Stem Cells[Title/Abstract]) OR (Adipose Derived Mesenchymal Stem Cells[Title/Abstract]) OR (Adipose Tissue-Derived Mesenchymal Stem Cell[Title/Abstract]) OR (Adipose Tissue Derived Mesenchymal Stem Cell[Title/Abstract]) OR (Adipose Tissue-Derived Mesenchymal Stem Cells[Title/Abstract]) OR (Adipose Tissue Derived Mesenchymal Stem Cells[Title/Abstract]) OR (Mesenchymal Stem Cells, Adipose-Derived[Title/Abstract]) OR (Mesenchymal Stem Cells, Adipose Derived[Title/Abstract]) OR (Adipose Tissue-Derived Mesenchymal Stromal Cells[Title/Abstract]) OR (Adipose Tissue Derived Mesenchymal Stromal Cells[Title/Abstract]) OR (Adipose-Derived Mesenchymal Stromal Cells[Title/Abstract]) OR (Adipose Derived Mesenchymal Stromal Cells[Title/Abstract]) OR (Adipose-Derived Mesenchymal Stem Cell[Title/Abstract]) OR (Adipose Derived Mesenchymal Stem Cell[Title/Abstract]) OR (Mesenchymal Stromal Cells[Title/Abstract]) OR (Stromal Cell, Mesenchymal[Title/Abstract]) OR (Stromal Cells, Mesenchymal[Title/Abstract]) OR (Mesenchymal Stromal Cell[Title/Abstract]) OR (Multipotent Mesenchymal Stromal Cells[Title/Abstract]) OR (Mesenchymal Stromal Cells, Multipotent[Title/Abstract]) OR (Multipotent Mesenchymal Stromal Cell[Title/Abstract]) OR (Mesenchymal Progenitor Cell[Title/Abstract]) OR (Mesenchymal Progenitor Cells[Title/Abstract]) OR (Progenitor Cell, Mesenchymal[Title/Abstract]) OR (Progenitor Cells, Mesenchymal[Title/Abstract]) OR (Wharton Jelly Cells[Title/Abstract]) OR (Wharton's Jelly Cells[Title/Abstract]) OR (Wharton's Jelly Cell[Title/Abstract]) OR (Whartons Jelly Cells[Title/Abstract]) | 85972 |
| #4 | "Osteoarthritis, Knee"[Mesh] OR (Knee Osteoarthritides) OR (Knee Osteoarthritis) OR (Osteoarthritis of Knee) OR (Osteoarthritis of the Knee) OR (Knee Arthrosis) OR (Knee Arthroses) OR (gonarthrosis[Title/Abstract]) | 91595 |
| #5 | #1 and (#2 OR #3) and #4 | 372 |

# Source: EMBASE

**Starting date:**

**Searched on:** March 24, 2025

**Results:** 302

| **Search** | **Query** | **Results** |
| --- | --- | --- |
| #1 | 'knee osteoarthritis'/exp OR 'Knee Osteoarthritides':ti,ab,kw OR 'Knee Osteoarthritis':ti,ab,kw OR 'Osteoarthritis of Knee':ti,ab,kw OR 'Osteoarthritis of the Knee':ti,ab,kw OR 'Knee Arthrosis':ti,ab,kw OR 'Knee Arthroses':ti,ab,kw OR 'gonarthrosis':ti,ab,kw OR 'femorotibial arthrosis':ti,ab,kw OR 'knee joint osteoarthritis':ti,ab,kw OR 'knee joint arthrosis':ti,ab,kw OR 'knee osteo-arthritis':ti,ab,kw OR 'knee osteo-arthrosis':ti,ab,kw OR 'knee osteoarthrosis':ti,ab,kw OR 'osteoarthritis, knee':ti,ab,kw OR 'osteoarthrosis, knee':ti,ab,kw OR 'arthrosis, knee':ti,ab,kw | 55468 |
| #2 | 'mesenchymal stem cell'/exp OR 'mesenchymal stem cells':ti,ab,kw OR 'stem cell, mesenchymal':ti,ab,kw OR 'stem cells, mesenchymal':ti,ab,kw OR 'mesenchymal stem cell':ti,ab,kw OR 'bone marrow mesenchymal stem cells':ti,ab,kw OR 'bone marrow mesenchymal stem cell':ti,ab,kw OR 'bone marrow stromal cells':ti,ab,kw OR 'bone marrow stromal cell':ti,ab,kw OR 'bone marrow stromal cells, multipotent':ti,ab,kw OR 'multipotent bone marrow stromal cell':ti,ab,kw OR 'multipotent bone marrow stromal cells':ti,ab,kw OR 'adipose-derived mesenchymal stem cells':ti,ab,kw OR 'adipose derived mesenchymal stem cells':ti,ab,kw OR 'adipose tissue-derived mesenchymal stem cell':ti,ab,kw OR 'adipose tissue derived mesenchymal stem cell':ti,ab,kw OR 'adipose tissue-derived mesenchymal stem cells':ti,ab,kw OR 'adipose tissue derived mesenchymal stem cells':ti,ab,kw OR 'mesenchymal stem cells, adipose-derived':ti,ab,kw OR 'mesenchymal stem cells, adipose derived':ti,ab,kw OR 'adipose tissue-derived mesenchymal stromal cells':ti,ab,kw OR 'adipose tissue derived mesenchymal stromal cells':ti,ab,kw OR 'adipose-derived mesenchymal stromal cells':ti,ab,kw OR 'adipose derived mesenchymal stromal cells':ti,ab,kw OR 'adipose-derived mesenchymal stem cell':ti,ab,kw OR 'adipose derived mesenchymal stem cell':ti,ab,kw OR 'mesenchymal stromal cells':ti,ab,kw OR 'stromal cell, mesenchymal':ti,ab,kw OR 'stromal cells, mesenchymal':ti,ab,kw OR 'mesenchymal stromal cell':ti,ab,kw OR 'multipotent mesenchymal stromal cells':ti,ab,kw OR 'mesenchymal stromal cells, multipotent':ti,ab,kw OR 'multipotent mesenchymal stromal cell':ti,ab,kw OR 'mesenchymal progenitor cell':ti,ab,kw OR 'mesenchymal progenitor cells':ti,ab,kw OR 'progenitor cell, mesenchymal':ti,ab,kw OR 'progenitor cells, mesenchymal':ti,ab,kw OR 'wharton jelly cells':ti,ab,kw OR 'whartons jelly cell':ti,ab,kw OR 'whartons jelly cells':ti,ab,kw OR 'bone marrow stromal stem cells':ti,ab,kw | 136002 |
| #3 | 'randomized controlled trial'/exp OR 'randomized controlled trial':ti,ab,kw OR randomized:ti,ab,kw OR placebo:ti,ab,kw OR 'clinical trial'/de OR 'randomized controlled trial'/de OR 'randomization'/de OR 'single blind procedure'/de OR 'double blind procedure'/de OR 'crossover procedure'/de OR 'placebo'/de OR 'prospective study'/de OR 'randomi?ed controlled' NEXT/1 trial* OR rct OR 'randomly allocated' OR 'allocated randomly' OR 'random allocation' OR allocated NEAR/2 random OR single NEXT/1 blind* OR double NEXT/1 blind* OR (treble OR triple) NEAR/1 blind* OR placebo* | 3375345 |
| #5 |  | 302 |

# Source: Scopus

**Starting date:**

**Searched on:** March 24, 2025

**Results:** 276

| **Search** | **Query** |  |
| --- | --- | --- |
| #1 | TITLE-ABS-KEY ("Knee Osteoarthritis" OR "Knee Osteoarthritides" OR "Osteoarthritis of Knee" OR "Osteoarthritis of the Knee" OR "Knee Arthrosis" OR "Knee Arthroses" OR "osteoarthritis,knee" OR "Gonarthrosis") | 53995 |
| #2 | TITLE-ABS-KEY ("Mesenchymal Stem Cells") OR TITLE-ABS-KEY ("Stem Cell, Mesenchymal") OR TITLE-ABS-KEY ("Stem Cells, Mesenchymal") OR TITLE-ABS-KEY ("Mesenchymal Stem Cell") OR TITLE-ABS-KEY ("Bone Marrow Mesenchymal Stem Cells") OR TITLE-ABS-KEY ("Bone Marrow Mesenchymal Stem Cell") OR TITLE-ABS-KEY ("Bone Marrow Stromal Cells") OR TITLE-ABS-KEY ("Bone Marrow Stromal Cell") OR TITLE-ABS-KEY ("Bone Marrow Stromal Cells, Multipotent") OR TITLE-ABS-KEY ("Multipotent Bone Marrow Stromal Cell") OR TITLE-ABS-KEY ("Multipotent Bone Marrow Stromal Cells") OR TITLE-ABS-KEY ("Bone Marrow Stromal Stem Cells") OR TITLE-ABS-KEY ("Adipose-Derived Mesenchymal Stem Cells") OR TITLE-ABS-KEY ("Adipose Derived Mesenchymal Stem Cells") OR TITLE-ABS-KEY ("Adipose Tissue-Derived Mesenchymal Stem Cell") OR TITLE-ABS-KEY ("Adipose Tissue Derived Mesenchymal Stem Cell") OR TITLE-ABS-KEY ("Adipose Tissue-Derived Mesenchymal Stem Cells") OR TITLE-ABS-KEY ("Adipose Tissue Derived Mesenchymal Stem Cells") OR TITLE-ABS-KEY ("Mesenchymal Stem Cells, Adipose-Derived") OR TITLE-ABS-KEY ("Mesenchymal Stem Cells, Adipose Derived") OR TITLE-ABS-KEY ("Adipose Tissue-Derived Mesenchymal Stromal Cells") OR TITLE-ABS-KEY ("Adipose Tissue Derived Mesenchymal Stromal Cells") OR TITLE-ABS-KEY ("Adipose-Derived Mesenchymal Stromal Cells") OR TITLE-ABS-KEY ("Adipose Derived Mesenchymal Stromal Cells") OR TITLE-ABS-KEY ("Adipose-Derived Mesenchymal Stem Cell") OR TITLE-ABS-KEY ("Adipose Derived Mesenchymal Stem Cell") OR TITLE-ABS-KEY("Adipose Tissue Derived Mesenchymal Stromal Cell") OR TITLE-ABS-KEY("Adipose Tissue Derived Mesenchymal Stromal Cell") OR TITLE-ABS-KEY ("Mesenchymal Stromal Cells") OR TITLE-ABS-KEY ("Stromal Cell, Mesenchymal") OR TITLE-ABS-KEY ("Stromal Cells, Mesenchymal") OR TITLE-ABS-KEY ("Mesenchymal Stromal Cell") OR TITLE-ABS-KEY ("Multipotent Mesenchymal Stromal Cells") OR TITLE-ABS-KEY ("Mesenchymal Stromal Cells, Multipotent") OR TITLE-ABS-KEY ("Multipotent Mesenchymal Stromal Cell") OR TITLE-ABS-KEY ("Mesenchymal Progenitor Cell") OR TITLE-ABS-KEY ("Mesenchymal Progenitor Cells") OR TITLE-ABS-KEY ("Progenitor Cell, Mesenchymal") OR TITLE-ABS-KEY ("Progenitor Cells, Mesenchymal") OR TITLE-ABS-KEY ("Wharton Jelly Cells") OR TITLE-ABS-KEY ("Wharton's Jelly Cells") OR TITLE-ABS-KEY ("Wharton's Jelly Cell") OR TITLE-ABS-KEY ("Whartons Jelly Cells") | 126837 |
| #3 | TITLE-ABS-KEY ( randomized AND controlled AND trial ) OR TITLE-ABS-KEY ( randomized ) OR TITLE-ABS-KEY ( placebo ) | 1706464 |
| #4 | #1 AND #2 AND #3 | 276 |

# Source: Web of science

**Starting date:**

**Searched on:** March 24, 2025

**Results:** 550

| **Search** | **Query** | **Results** |
| --- | --- | --- |
| #1 | TS=(Mesenchymal Stem Cells OR Stem Cell, Mesenchymal OR Stem Cells, Mesenchymal OR Mesenchymal Stem Cell OR Bone Marrow Mesenchymal Stem Cells OR Bone Marrow Mesenchymal Stem Cell OR Bone Marrow Stromal Cells OR Bone Marrow Stromal Cell OR Bone Marrow Stromal Cells, Multipotent OR Multipotent Bone Marrow Stromal Cell OR Multipotent Bone Marrow Stromal Cells OR Adipose‑Derived Mesenchymal Stem Cells OR Adipose Derived Mesenchymal Stem Cells OR Adipose Tissue‑Derived Mesenchymal Stem Cell OR Adipose Tissue Derived Mesenchymal Stem Cell OR Adipose Tissue‑Derived Mesenchymal Stem Cells OR Adipose Tissue Derived Mesenchymal Stem Cells OR Mesenchymal Stem Cells, Adipose‑Derived OR Mesenchymal Stem Cells, Adipose Derived OR Adipose Tissue‑Derived Mesenchymal Stromal Cells OR Adipose‑Derived Mesenchymal Stromal Cells OR Adipose Tissue Derived Mesenchymal Stromal Cells OR Adipose Derived Mesenchymal Stromal Cells OR Adipose‑Derived Mesenchymal Stem Cell OR Adipose Derived Mesenchymal Stem Cell OR Mesenchymal Stromal Cells OR Stromal Cell, Mesenchymal OR Stromal Cells, Mesenchymal OR Mesenchymal Stromal Cell OR Multipotent Mesenchymal Stromal Cells OR Mesenchymal Stromal Cells, Multipotent OR Multipotent Mesenchymal Stromal Cell OR Mesenchymal Progenitor Cells OR Mesenchymal Progenitor Cell OR Progenitor Cell, Mesenchymal OR Progenitor Cells, Mesenchymal OR Wharton Jelly Cells OR Wharton’s Jelly Cells OR Wharton’s Jelly Cell OR wharton Jelly Cells OR Bone Marrow Stromal Stem Cells OR Adipose Tissue‑Derived Mesenchymal Stromal Cell OR Adipose Tissue Derived Mesenchymal Stromal Cell) | 260029 |
| #2 | TS=((Knee Osteoarthritis) OR (Osteoarthritis, Knee) OR (Knee osteoarthritises) OR (Osteoarthritis of Knee) OR (Osteoarthritis of the Knee) OR (Knee Arthrosis) OR (Knee arthrosis) OR (Gonarthrosis)) | 106646 |
| #3 | TS=(randomized controlled trial OR controlled clinical trial OR randomized OR placebo OR randomly) | 3114307 |
| #4 | #1 AND #2 AND #3 | 550 |

# Source: Cochrane Central Register of Controlled Trials

**Starting date:**

**Searched on:** March 24, 2025

**Results:** 182

| **Search** | **Query** | **Results** |
| --- | --- | --- |
| #1 | Randomized Controlled Trial OR Double-Blind Method OR Placebos OR Random Allocation | 1278285 |
| #2 | MeSH descriptor: [Osteoarthritis, Knee] explode all trees | 6975 |
| #3 | (Knee Osteoarthritides):ti,ab,kw OR (Knee Osteoarthritis):ti,ab,kw OR (Knee Arthroses):ti,ab,kw OR (Osteoarthritis of Knee):ti,ab,kw OR (Osteoarthritis of the Knee):ti,ab,kw OR (Knee Arthrosis):ti,ab,kw | 18765 |
| #4 | MeSH descriptor: [Mesenchymal Stem Cells] explode all trees | 360 |
| #5 | (Mesenchymal Stem Cells):ti,ab,kw OR (Stem Cell, Mesenchymal):ti,ab,kw OR (Stem Cells, Mesenchymal):ti,ab,kw OR (Mesenchymal Stem Cell):ti,ab,kw OR (Bone Marrow Mesenchymal Stem Cells):ti,ab,kw OR (Bone Marrow Mesenchymal Stem Cell):ti,ab,kw OR (Bone Marrow Stromal Cells):ti,ab,kw OR (Bone Marrow Stromal Cell):ti,ab,kw OR (Bone Marrow Stromal Cells, Multipotent):ti,ab,kw OR (Multipotent Bone Marrow Stromal Cell):ti,ab,kw OR (Multipotent Bone Marrow Stromal Cells):ti,ab,kw OR (Adipose-Derived Mesenchymal Stem Cells):ti,ab,kw OR (Adipose Derived Mesenchymal Stem Cells):ti,ab,kw OR (Adipose Tissue-Derived Mesenchymal Stem Cell):ti,ab,kw OR (Adipose Tissue Derived Mesenchymal Stem Cell):ti,ab,kw OR (Adipose Tissue-Derived Mesenchymal Stem Cells):ti,ab,kw OR (Adipose Tissue Derived Mesenchymal Stem Cells):ti,ab,kw OR (Mesenchymal Stem Cells, Adipose-Derived):ti,ab,kw OR (Mesenchymal Stem Cells, Adipose Derived):ti,ab,kw OR (Adipose Tissue-Derived Mesenchymal Stromal Cells):ti,ab,kw OR (Adipose Tissue Derived Mesenchymal Stromal Cells):ti,ab,kw OR (Adipose-Derived Mesenchymal Stromal Cells):ti,ab,kw OR (Adipose Derived Mesenchymal Stromal Cells):ti,ab,kw OR (Adipose-Derived Mesenchymal Stem Cell):ti,ab,kw OR (Adipose Derived Mesenchymal Stem Cell):ti,ab,kw OR (Mesenchymal Stromal Cells):ti,ab,kw OR (Stromal Cell, Mesenchymal):ti,ab,kw OR (Stromal Cells, Mesenchymal):ti,ab,kw OR (Mesenchymal Stromal Cell):ti,ab,kw OR (Multipotent Mesenchymal Stromal Cells):ti,ab,kw OR (Mesenchymal Stromal Cells, Multipotent):ti,ab,kw OR (Multipotent Mesenchymal Stromal Cell):ti,ab,kw OR (Mesenchymal Progenitor Cell):ti,ab,kw OR (Mesenchymal Progenitor Cells):ti,ab,kw OR (Progenitor Cells, Mesenchymal):ti,ab,kw OR (Progenitor Cell, Mesenchymal):ti,ab,kw OR (Wharton Jelly Cells):ti,ab,kw OR (Wharton's Jelly Cells):ti,ab,kw OR (Wharton's Jelly Cell):ti,ab,kw OR (Whartons Jelly Cells):ti,ab,kw OR (Bone Marrow Stromal Stem Cells):ti,ab,kw | 2606 |
| #6 | #1 AND (#2 OR #3) AND (#4 OR #5) | 182 |
