# Supplementary material for: Contextual effects of mesenchymal stem cell injections for knee osteoarthritis: systematic review and meta-analysis of randomized controlled trials
Source: Front Med (Lausanne). 2025 Sep 17;12:1636181. doi: 10.3389/fmed.2025.1636181 (PMC12487426; doi:10.3389/fmed.2025.1636181)
Supplement: Supplementary file 3 [file Table_3.docx]

| Title | Classification | Reason |
| --- | --- | --- |
| Evaluation of mesenchymal stem cells in the treatment of knee osteoarthritis – A randomised Controlled Trial | No placebo | MSC + Conservative Treatment vs Conservative Treatment Conservative therapy: analgesic medications (e.g., NSAIDs)/weight management and nutritional guidance/physical therapy (exercise program directed by a physical therapist or physician)/biomechanical adjustments (e.g., braces or orthotics) Interventional therapies:original conservative measures (e.g., medications, exercise) may be allowed to continue, but adjustments need to be communicated to the investigator (e.g., analgesics for postoperative management) |
| The evaluation of allogeneic adipose derived mesenchymal stem cells as a treatment for symptomatic knee osteoarthritis, specifically evaluating safety, effects on knee pain, function and structure, and quality of life in knee osteoarthritis patients | duplicate document | Syn: Safety, tolerability and efficacy of intra-articular Progenza in knee osteoarthritis: a randomized double-blind placebo-controlled singleascending dose study This article is registered: ACTRN12615000439549; same as incorporated into the |
| Stem cell therapy in knee osteoarthritis | Ongoing trials | Registration No.: ACTRN12620000870954; Recent developments: 20240422 Official Website: https://trialsearch.who.int/Trial2.aspx?TrialID=ACTRN12620000870954 Sample size: 320; PROTOCOL=134Degree of fit: High fit |
| Matrix-induced autologous mesenchymal stem cell implantation versus matrix-induced autologous chondrocyte implantation in the treatment of chondral defects of the knee: a 2-year randomized study | No placebo | Matrix-induced autologous mesenchymal stem cell implantation (m-AMI) Matrix-induced autologous chondrocyte implantation (m-ACI) Effectiveness in the treatment of isolated knee cartilage defects |
| Intra-articular injection of culture-expanded mesenchymal stem cells with or without addition of platelet-rich plasma is effective in decreasing pain and symptoms in knee osteoarthritis: a controlled, double-blind clinical trial | No placebo | MSCs group (n=16): received culture-amplified autologous bone marrow-derived MSCs injection; MSCs+PRP group (n=14): received culture-expanded autologous bone marrow-derived MSCs combined with PRP injections; Corticosteroid group (n=17): received intra-articular corticosteroid injections. |
| Bone marrow aspirate concentrate injections provide similar results versus viscosupplementation up to 24 months of follow-up in patients with symptomatic knee osteoarthritis. A randomized controlled trial | No placebo | The same patient was treated with BMAC and HA in both knees, and both knees received different treatments |
| Safety and efficacy of placental mesenchymal stromal cells-derived extracellular vesicles in knee osteoarthritis: a randomized, triple-blind, placebo-controlled clinical trial | Interventions other than MSC therapy only | Study for MSC secretion |
| Treatment of knee osteoarthritis with intra-articular injection of allogeneic adipose-derived stem cells (ADSCs) ELIXCYTE®: a phase I/II, randomized, active-control, single-blind, multiple-center clinical trial | No placebo | Allogeneic adipose-derived stem cells (ADSCs) were compared with HA;Intervention group: Using allogeneic adipose-derived stem cells (ADSCs) ELIXCYTE®by single intra-articular injection, divided into three groups: Low dose group(16 x 10*6 cells) Medium dose group(32 x 10*6 cells) High dose group(64 x 10*6 cells).Control group: the cells were injected with hyaluronic acid (HA)(Hya Joint Plus, 3 mL) for single intra-articular injectionThe intervention group did not use HA, but was compared to an independent HA control group with the aim of comparing the efficacy of the two |
| A clinical trial to study the effect of different doses of stem cells in Osteoarthritis | Ongoing trials | CTRI/2011/07/001891; https://www.ctri.nic.in/Clinicaltrials/advsearch2.php https://trialsearch.who.int/Trial2.aspx?TrialID=CTRI/2011/07/001891 http://www.ctri.nic.in/Clinicaltrials/pmaindet2.php?trialid=2734Fit: very high Drug/treatment: allogeneic bone marrow-derived mesenchymal stem cellsControl: placebo (Plasmalyte-A, an electrolyte solution) |
| A clinical trial to evaluate the effects of stem cells in patients with Osteoarthritis of the Knee joint | No placebo | Intervention group: single injection of 25 million BMMSCs + hyaluronic acid.Control group: single injection of placebo + hyaluronic acid.STUDY CORE: To validate the efficacy and safety of BMMSCs in improving knee osteoarthritis function, relieving symptoms, and protecting cartilage, and to challenge the limitations of conventional treatment. CTRI/2018/09/015785 (exclude 3 after reading full article) |
| Evaluating Synergistic Effects of Hyaluronic Acid, Human Umbilical Cord-Derived Mesenchymal Stem Cells, and Growth Hormones in Knee Osteoarthritis: A Multi-Arm Randomized Trial | No placebo | HA-only group, HA combined with hUC-MSCs group, HA combined with growth hormone group, and combined HA, hUC-MSCs, and growth hormone group NCT03800810 (exclusion 4 after reading the full article) |
| Allogeneic mesenchymal stromal cell (MSC) therapy for knee osteoarthritis (OA): A phase I/II randomized controlled trial | Non-primary research | Conference Abstracts. 25th Annual Meeting of the International Society for Cellular Therapy, ISCT 2017 Intervention group. MSC1 group (single UC-MSC injection) MSC2 group (two UC-MSC injections) Control group: Positive control group (HA group) (received hyaluronic acid injection) The abstract clearly states that the control group receives HA injections while the intervention group receives only UC-MSC treatment and that the two interventions do not cross each other; |
| A phase 2b Study Evaluating the Efficacy of a Single Injection Autologous Adipose Derived Mesenchymal Stromal Cells in Patients with Knee Osteoarthritis | Ongoing trials | EudraCT Number: 2015-002125-19;Fit: very high;data not available https://www.clinicaltrialsregister.eu/ctr-search/search?query=eudract_number:2015-002125-19 |
| A Randomized, Double Blind, Two Arms, Controlled Phase I/II Safety and Efficacy Study on MesoCellA-Ortho Tissue Engineered Product Intraarticularly Administrated in Adult Patients with Osteoarthrosis | Ongoing trials | Fit: high (unknown for intervention group with or without HA)Registry number: EUCTR2019-005000-17-PL; last update: 20240312Intervention group: autologous adipose-derived mesenchymal stem cells combined with hyaluronic acid intra-articular injectionControl group: hyaluronic acid intra-articular injection onlyDetails unknown, no data, no articlehttps://trialsearch.who.int/Trial2.aspx?TrialID=EUCTR2019-005000-17-PL |
| Adipose-derived mesenchymal stem cell therapy in the treatment of knee osteoarthritis: a randomized controlled trial | No placebo | Control group: maintained on conventional conservative treatmentincluding: medications: analgesics (e.g. NSAIDs), supplements such as glucosamine sulfate, and non-pharmacological interventions, no invasive interventions.Single injection group: received one intra-articular injection of autologousadipose-derived mesenchymal stem cells (ADMSCs) Two injection group: received oneinjection ofthe same dose ofADMSCsat baseline and oneinjectionafter 6 months ACTRN12614000814673 |
| Angiopoietin-like 3-derivative LNA043 for cartilage regeneration in osteoarthritis: a randomized phase 1 trial | Interventions other than MSC therapy only | Phenotypic screening of human mesenchymal stem cells reveals a derivative derived from angiopoietin-like protein 3 (ANGPTL3)-LNA043 Experimental group: received LNA043 injection (3/4 of the randomized proportion) Control group (placebo group): received placebo injection (1/4 of the randomized proportion) |
| One-step surgery with multipotent stem cells and Hyaluronan-based scaffold for the treatment of full-thickness chondral defects of the knee in patients older than 45 years | Non-RCT | Control group: consisted of patients younger than 45 years of age and was used to compare treatment outcomes with patients older than 45 years of age (study group). Experimental group: all patients received the same stem cell combined with stent therapy, and the comparison focused on the difference in efficacy between the different age groups. This is a prospective cohort study (Level II evidence) and is an observational intervention study, not a randomized controlled trial (RCT) |
| The comparison of knee osteoarthritis treatment with single-dose bone marrow-derived mononuclear cells vs. hyaluronic acid injections | Interventions other than MSC therapy only | Control group: Patients treated with hyaluronic acid (three injections once a week). Experimental group: Patients treated with a single injection of bone marrow-derived single nucleated cells. Prospective Randomized Clinical Trial |
| Efficacy and safety of adult human bone marrow-derived, cultured, pooled, allogeneic mesenchymal stromal cells (Stempeucel®): preclinical and clinical trial in osteoarthritis of the knee joint | No placebo | n=60 NCT01453738 (exclusion 1 after reading full text) DOI: 10.1186/s13075-016-1195-7 |
| Mesenchymal Stem/Stromal Cells: ALLOGENEIC, OFF THE SHELF, POOLED, BM - MSCS (STEMPEUCEL®) – A POTENTIAL BREAK THROUGH THERAPY FOR GRADE II AND III OSTEOARTHRITIS KNEE | No placebo | Placebo + hyaluronic acid, MSC + hyaluronic acidCTRI/2018/09/015785(with 42: A clinical trial to evaluate the effects of stem cells in patients with Osteoarthritis of the Knee joint, and the article is a conference abstract) |
| Efficacy and Safety of Stempeucel in Osteoarthritis of the Knee: A Phase 3 Randomized, Double-Blind, Multicenter, Placebo-Controlled Study | No placebo | Placebo group (control group): Patients receive a placebo injection followed by hyaluronic acid. Experimental group: Patients receive an intra-articular injection of bone marrow-derived MSC (25 million cells) followed by hyaluronic acid. CTRI/2018/09/015785, same as 78: Mesenchymal Stem/Stromal Cells: ALLOGENEIC, OFF THE SHELF, POOLED, BM - MSCS (STEMPEUCEL®) - A POTENTIAL BREAK THROUGH THERAPY for GRADE II and III OSTEOARTHRITIS KNEE |
| Randomized control trial of mesenchymal stem cells versus hyaluronic acid in patients with knee osteoarthritis – A Hong Kong pilot study | No placebo | MSC vs HA alone This trial utilized an active control design of "MSC injection vs HA injection" rather than a background control of "same HA or PRP with/without MSC". The two groups were completely different in terms of injection composition (MSC vs HA), there was no way to provide a common baseline for the "additive effect of MSC" (i.e., there was no guarantee that the only difference would be the presence or absence of the MSC component), and there was no placebo control or dummy injections to subtract the procedural effect of the injections themselves. Therefore, if you want to strictly screen for studies that "use the same injection background for both control and intervention, and differ only in MSC content," this is similar to other previous "MSC vs HA" head-to-head designs, and is recommended for exclusion. If in the future you want to more loosely include all "MSC vs. any injection" trials before stratification, you can selectively include them, but it is not recommended under the current design concept. |
| The effect of mesenchymal stem cells on osteoarthritis recovery | Ongoing trials | Control group: Received intra-articular knee injections containing only hyaluronic acid (5 mL). EXPERIMENTAL GROUP: Receive intra-knee injections of 40 million umbilical cord-derived MSC loaded in 5 mL of hyaluronic acid.Fit: very high;IRCT20210103049925N1; target sample size: 40 https://trialsearch.who.int/Trial2.aspx?TrialID=IRCT20210103049925N1 |
| Mesenchymal stem cell injection in patients with knee osteoarthritis compared to a randomized controlled trial | Ongoing trials | Registration number: IRCT20210307050611N1;Contact website: https://trialsearch.who.int/Trial2.aspx?TrialID=IRCT20210307050611N1Control group: knee care training in activities of daily livingIntervention group: 8 million adipose-derived stem cells (counted as relatively pure MSC) containing 2 ml of salineLast refreshed 20210522; no data status,fit: high |
| Effect of administration of mesenchymal stem cells on cartilage recovery and knee function in patients with Knee Osteoarthritis | No placebo | Not a placebo control group but a no treatment group The control group was simply the "no MSCs treatment" group and did not receive any alternative intervention or placebo, which the original article did not say!Read the full article and exclude 33 |
| Safety and efficacy of allogenic placental mesenchymal stem cells for treating knee osteoarthritis: a pilot study | Non-primary research | An incorporated abstract of the conference of the same name |
| Effects of intra-articular autologous mesenchymal stem cell injection under ultrasonogram guidance in patients with osteoarthritis knee | Non-RCT | No control group, non-RCT, excluded after reading the full article6 |
| Intra-articular Injection of Mesenchymal Stem Cells After High Tibial Osteotomy in Osteoarthritic Knee: Two-Year Follow-up of Randomized Control Trial | No placebo | Control group: group of patients who underwent MOWHTO surgery only (n = 13). Experimental group: group of patients who underwent MOWHTO surgery combined with ADMSC intra-articular injection (n = 13) |
| Infrapatellar fat pad-derived mesenchymal stem cell therapy for knee osteoarthritis | No placebo | Experimental group: received arthroscopic debridement + PRP + MSCs injectionControl group: received arthroscopic debridement + PRP The aim of this study was to investigate the safety and potential efficacy of intra-articular injection of autologous mesenchymal stem cells (MSCs) derived from infrapatellar fat pad in patients with osteoarthritis of the knee (OA) in improvingosteoarthritis (OA) patients and their potential efficacy in improving pain and knee function. The investigators wanted to verify whether the additional addition of infrapatellar fat pad-derived MSC injections resulted in better clinical outcomes in terms of pain reduction and improved function compared with arthroscopic debridement combined with platelet-rich plasma (PRP) injections alone. |
| Comparative outcomes of open-wedge high tibial osteotomy with platelet-rich plasma alone or in combination with mesenchymal stem cell treatment: a prospective study | No placebo | Control group: HTO+PRP (PRP alone group, n = 23). Experimental group: HTO + PRP + MSC (MSC-PRP group, n = 21) Prospective comparative observational study Purpose: Whether MSC combined with PRP can further optimize clinical outcomes (pain relief, improved function) and promote cartilage repair after HTO To compare the results of two regimens, PRP injection alone and PRP + mesenchymal stem cell (MSC) treatment, after an orthopedic (open high tibial osteotomy, HTO) knee osteotomy (Varus deformity). |
| Phase II multicenter randomized controlled clinical trial on the efficacy of intra-articular injection of autologous bone marrow mesenchymal stem cells with platelet rich plasma for the treatment of knee osteoarthritis | No placebo | Control group: Patients who received PRGF® alone. Experimental group: Patients receiving BM-MSCs in combination with PRGF® injection. EudraCT: 2011-006036-23 NCT02365142 (exclude 8 after reading the full article) |
| Intra-articular injection of two different doses of autologous bone marrow mesenchymal stem cells versus hyaluronic acid in the treatment of knee osteoarthritis: multicenter randomized controlled clinical trial (phase I/II) | No placebo | Control group: patients who received hyaluronic acid injections only NCT02123368.EudraCT: 2009-017624-72 (exclude 9 after reading full text) Experimental group: patients who received hyaluronic acid combined with BM-MSCs injections, which were divided into a low-dose group (10×10^6 cells) and a high-dose group (100×10^6 cells). |
| The Efficacy Of Acupuncture Combined With Mesenchymal Stem Cell Injection In The Treatment Of Osteoarthritis Of The Knee | No placebo | Acupuncture + MSC, MSC |
| Efficacy and cost-effectiveness of Stem Cell injections for symptomatic relief and strUctural improvement in people with Tibiofemoral knee OsteoaRthritis: protocol for a randomised placebo-controlled trial (the SCUlpTOR trial) | Ongoing trials | Fit: High(MSC with 0.9% NaCl solution), n=440 Registry Number: ACTRN12620000870954 Last update: 20240417 |
| Intra-articular injections of allogeneic human adipose-derived mesenchymal progenitor cells in patients with symptomatic bilateral knee osteoarthritis: a Phase I pilot study | No placebo | This study was a dose-exploration trial in which patients were randomized into three different dose groups (low, medium, and high dose) The three dose groups were not set up in the traditional sense of a placebo or control group. |
| Treatment of knee osteoarthritis with intra-articular injection of autologous adipose-derived mesenchymal progenitor cells: a prospective, randomized, double-blind, active-controlled, phase IIb clinical trial | No placebo | MSC vs. HA, very good trial design Controls were active treatments and lacked common ground: the control group received HA injections while the intervention group received MSC injections, and both did not share the same underlying treatment. Differences between groups included not only the presence or absence of MSC, but also the presence or absence of HA treatment, so it is not possible to attribute differences in outcome purely to theeffectof MSC |
| Treatment with human adipose-derived mesenchymal stem cells for knee OA: evidences from a randomized and double-blinded phase I/II a clinical trial | No placebo | All dose groups, no placebo, experimental groups for different doses: low, medium and high dose haMSCs groups |
| Allogeneic, Off The Shelf, Bone Marrow derived, Pooled, Mesenchymal Stromal Cells - A Potential Break through Therapy for Grade II & III Osteoarthritis Knee Management | Interventions other than MSC therapy only | Experimental group (treatment group): patients who received stempeucel® intra-articular injection (25 million cells) followed by 20 mg hyaluronic acid. Control group: Patients who received saline injection (placebo injection) followed by 20 mg hyaluronic acid. CTRI/2018/09/015785, same as 78: Mesenchymal Stem/Stromal Cells: ALLOGENEIC, OFF THE SHELF, POOLED, BM - MSCS (STEMPEUCEL®) - A POTENTIAL BREAK THROUGH THERAPY for GRADE II and III OSTEOARTHRITIS KNEE |
| A Phase I Dose-Escalation Clinical Trial to Assess the Safety and Efficacy of Umbilical Cord-Derived Mesenchymal Stromal Cells in Knee Osteoarthritis | No placebo | Three dose groups |
| Umbilical Cord-Derived Mesenchymal Stromal Cells (MSCs) for Knee Osteoarthritis: Repeated MSC Dosing Is Superior to a Single MSC Dose and to Hyaluronic Acid in a Controlled Randomized Phase I/II Trial | No placebo | HA group (n = 8): received hyaluronic acid injections at baseline and at 6 months;MSC-1 group (n = 9): received a single injection of 20 × 10*6 UC-MSCs at baseline;MSC-2 group (n = 9): received a single injection of 20 × 10*6 UC-MSCs at baseline and 6 months eachNo placebo.For group 1 vs. group 2 did they form a control at December? Same as 172, similar to 66 |
| Study to Compare Efficacy and Safety of Cartistem and Microfracture in Patients With Knee Articular Cartilage Injury | No placebo | NCT01041001 (joint), read more exclude 14 Comparison of Allogeneic Umbilical Cord Blood Mesenchymal Stem Cells (UCB-MSC) Implantation with 4% Hyaluronic Acid (HA) Complex and Microfracture Surgery |
| Allogeneic Mesenchymal Stem Cells for Osteoarthritis | No placebo | NCT01448434, MSC+HA vs placebo+HAFit: extremely high https://clinicaltrials.gov/study/NCT01448434 |
| Allogeneic Mesenchymal Stem Cells in Osteoarthritis | No placebo | All injected with a little HA, triple dose group vs placebo (balanced electrolytes), NCT01453738 Same (Efficacy and safety of adult human bone marrow-derived, cultured, pooled, allogeneic mesenchymal stromal cells (Stempeucel®): preclinical and clinical trial in osteoarthritis of the knee joint) |
| Treatment of Knee Osteoarthritis With Allogenic Mesenchymal Stem Cells | No placebo | NCT01586312, with HA in the control group (this trial was based on our previous results with autologous MSCs (ongoing trial NCT01183728)) The study clearly differentiated between the two treatment regimens: the MSC group received only injections of allogeneic MSCs, not combined with HA; No placebo34 (AI recommended exclusion) |
| Treatment of Knee Osteoarthritis by Intra-articular Injection of Bone Marrow Mesenchymal Stem Cells | No placebo | MSC+HA, HA; NCT02123368, with 125: Intra-articular injection of two different doses of autologous bone marrow mesenchymal stem cells versus hyaluronic acid in the treatment of knee osteoarthritis: multicenter randomized controlled clinical trial (phase I/II) |
| Clinical Trial of Autologous Adipose Tissue-Derived Mesenchymal Progenitor Cells (MPCs) Therapy for Knee Osteoarthritis | No placebo | NCT02162693, n=52Control group HA only; intervention group MSC only To assess safety: the safety of intra-articular injection of autologous adipose-derived mesenchymal precursor cells (haMPCs, trade name Re-Join®) for the treatment of knee osteoarthritis (OA). To assess efficacy: to compare the difference in efficacy between Re-Join® and the active control sodium vitrate (HA) in terms of reduction of patient pain, improvement of joint function, and cartilage repair over a 12-month follow-up period. |
| Treatment of Osteoarthritis by Intra-articular Injection of Bone Marrow Mesenchymal Stem Cells With Platelet Rich Plasma | No placebo | Group A (control group): PRGF only (existing biologic therapy with some known efficacy).Group B (experimental group): addition of MSCs to PRGF to verify whether it provides additional benefit. vs 124: Phase II multicenter randomized controlled clinical trial on the efficacy of intra-articular injection of autologous bone marrow mesenchymal stem cells with platelet rich plasma for the treatment of knee osteoarthritis Repeat |
| Clinical Trial of Allogenic Adipose Tissue-Derived Mesenchymal Progenitor Cells Therapy for Knee Osteoarthritis | No placebo | NCT02641860 Patients Receiving Different Doses of Allogeneic haMPCs (Low, Medium, and High Dose) Injections |
| A Study to Assess Safety and Efficacy of Umbilical Cord-derived Mesenchymal Stromal Cells in Knee Osteoarthritis | No placebo | NCT02580695 HA group: patients receiving hyaluronic acid treatment. MSC-1 group: patients who received a single umbilical cord-derived MSC injection. MSC-2 group: patients who received two umbilical cord-derived MSC injections. Similar to 155 & 66 |
| A Study Evaluating the Efficacy of a Single Injection Autologous Adipose Derived Mesenchymal Stromal Cells in Patients With Knee Osteoarthritis | Ongoing trials | Registry Number: NCT02838069; Current Status: Active, not recruitingFit status: high fit(test group is ASC, placebo control: placebo 0.5% glucose saline and 4.5% albumin) https://clinicaltrials.gov/study/NCT02838069 This article comes from a Springer book that describes the latest clinical trials and research advances in the field of orthopedics using stem cells to treat a variety of diseases p123-125 |
| The Comparison of Efficacy and Safety of the Mesenchymal Stem Cells From Adipose and Hyaluronic Acid | No placebo | Experimental group: group receiving intra-articular injections of adipose-derived mesenchymal stem cells (MSCs).Control group: group receiving hyaluronic acid (HA) injections (active control, i.e., current standard treatment) No placebo, NCT03357575; current status: unknown status (n=14); no data found on the internetFit: low;last update: 20171130 https://clinicaltrials.gov/study/NCT03357575 |
| The Effects of Stromal Vascular Fraction and Mesenchymal Stem Cells as Intra-articular Injection in Knee Joint Osteoarthritis | Ongoing trials | NCT03164083; current status: test aborted https://clinicaltrials.gov/study/NCT03164083Fit: High fit |
| Implantation of Allogenic Mesenchymal Stem Cell From Umbilical Cord Blood for Osteoarthritis Management | No placebo | Arm 1: Sodium vitrate (HA) aloneArm 2: HA + Umbilical cord-derived mesenchymal stem cells (hUC-MSC)Arm 3: HA + synthetic growth hormone (GH/Somatotropin)Arm 4: HA + GH + hUC-MSC (combination of all three)Sample size only 3 per group and artificial synovial fluidn=28, 51 knees (excluded 14 after reading full article) |
| Allogenic Adipose Tissue-Derived Mesenchymal Progenitor Cells for the Treatment of Knee Osteoarthritis | No placebo | NCT04208646, current status: completed, last update: 20230801Fit: highly composite doubtful Intervention: mesenchymal progenitor cells low dose group, mesenchymal progenitor cells high dose group; Control: no mesenchymal stem cells (unknown) https://clinicaltrials.gov/study/NCT04208646 |
| Clinical Study of Intra Articular Injection of Catholic MASTER Cell (Bone Marrow Derived Mesenchymal Stem Cell) in Knee Osteoarthritis | duplicate document | Syn: Intra-Articular Injection of Human Bone Marrow-Derived Mesenchymal Stem Cells in Knee Osteoarthritis: A Randomized, Double-Blind, Controlled Trial |
| A Phase 3 Study to Evaluate the Efficacy and Safety of JointStem in Treatment of Osteoarthritis | duplicate document | NCT03990805 Syn: Clinical Efficacy and Safety of the Intra-articular Injection of Autologous Adipose-Derived Mesenchymal Stem Cells for Knee Osteoarthritis: A Phase III, Randomized, Double-Blind, Placebo-Controlled Trial |
| RCT Mesenchymal Stem Cells Versus Hyaluronic Acid in OA Knee) | Ongoing trials | Group A: Mesenchymal stem cells (MSCs) injection group Group B: Hyaluronic acid (HA) injection group Group C: No injection group NCT04326985, current status: completed; last update: 20200518 https://clinicaltrials.gov/study/NCT04326985Group A vs. Group C control?No data available |
| Evaluation of Safety and Efficacy of Wharton's Jelly Compared to Hyaluronic Acid and Saline for Knee Osteoarthritis | Non-primary research | No. 25, NCT04711304 1. Intervention group (WJ group) Treatment: single intra-articular knee injection.Drug composition: GeneXSTEM® (BioIntegrate Inc.) product containing umbilical cord-derived Walden's Jelly.Dosage and Administration: 2 mL WJ diluted with 1:1 sterile saline for injection.Crossover design: switching to WJ treatment after 3 months was allowed (only for the original HA and saline groups). 2. Control group (HA group) Treatment: Single intra-articular knee injection.Drug composition: Monovisc® (Anika Therapeutics), a highly concentrated hyaluronic acid (22 mg/mL).Dose: 4 mL (standard clinical dosage).Mechanism of action: as a positive control to verify the known efficacy and safety of HA. 3. Placebo group (saline group) Treatment: single intra-articular knee injection.Drug composition: 4 mL of sterile saline.Purpose: To exclude the placebo effect and to assess the true efficacy of the intervention. |
| Allogenic Mesenchymal Stem Cell Intraarticular Injection for Knee Osteoarthritis Therapy | Ongoing trials | Fit: very high; Current status: hiring Last updated: 20240611 https://clinicaltrials.gov/study/NCT05933434 NCT05933434 |
| Efficacy and Safety of IxCell hUC-MSC-O in Patients With Knee Osteoarthritis | Ongoing trials | Fit: higher fit;NCT06716281; last updated: 20241206; https://clinicaltrials.gov/study/NCT06716281 |
| Treatment of knee osteoarthritis with autologous mesenchymal stem cells: a pilot study | No placebo | This study preliminarily confirmed the safety, feasibility and significant efficacy of autologous MSCs in the treatment of knee osteoarthritis through a single-group pilot design, but further validation of its superiority through a randomized controlled trial (RCT) is needed. |
| Umbilical Cord Mesenchymal Stem Cell Secretome Improves Clinical Outcomes and Changes Biomarkers in Knee Osteoarthritis | No placebo | UC-MSC secretion group (n=15): 2 mL of secretion group injected intra-articularly weekly for 5 weeks. HA group (n=15): same dose of HA injection. NCT05579665 |
| Human adipose-derived mesenchymal progenitor cells plus microfracture and hyaluronic acid for cartilage repair: a Phase IIa trial | Interventions other than MSC therapy only | A total of 30 patients suffering from cartilage defects in the knee joint of the medial femoral condyle were randomized into three groups: Arthroscopic microfracture surgery plus saline injection group Arthroscopic microfracture plus articular hyaluronic acid injection group Arthroscopic microfracture combined with intra-articular hyaluronic acid and haMPCs injection group  NCT02855073 |
| START Trial: randomized, controlled, open and parellel trial to evaluate the efficiency and safety of intra-articular infiltration of Autologous Bone Marrow Concentrate (BMC) or Mesenchymal Stem Cell (MSC) associated with Platelet Rich Plasma (PRP) in patients with primary knee osteoarthritis | Interventions other than MSC therapy only | Control group (Group 1): 64 cases received a single intra-articular injection of hyaluronic acid (Synvisc One, 6 mL) and were followed up for 12 months.Experimental group (PRP group, Group 2): 64 cases prepared platelet-rich plasma (PRP) by blood collection of 15 mL, divided into three injections (1-week interval), followed up for 12 months.Experimental group (CTM+PRP group, Group 3): 64 cases were collected about 100 mL of iliac bone marrow cells, laboratory processed for 15 days and then co-injected with PRP (the first injection of bone marrow cells + PRP, and the subsequent two injections of PRP only), followed up for 12 months.Experimental group (CMO+PRP group, Group 4): 64 cases of iliac bone marrow cells collected, processed quickly by a certified laboratory (about 2 hours), co-injected with PRP the next day (first injection of bone marrow cells + PRP, two subsequent injections of PRP only), followed up for 12 months.Ask if AI is wanted, CTM? CMO?Registry number: RBR-7qjcdd; no data statusFit: low |
| Cartilage regeneration and inflammation modulation in knee osteoarthritis following injection of allogeneic adipose-derived mesenchymal stromal cells: a phase II, triple-blinded, placebo controlled, randomized trial | duplicate document | 40 name Same: Allogeneic stem cell transplantation in knee joint osteoarthritis |
| Combination of Intra-Articular and Intraosseous Injections of Platelet Rich Plasma for Severe Knee Osteoarthritis: A Pilot Study | Non-RCT | A single-arm prospective pilot study (Pilot Study) was conducted to evaluate the clinical efficacy of a novel combination therapy (intra-articular injection + intra-osseous injection of platelet-rich plasma PRP) in patients with severe knee osteoarthritis (KOA). Read the full article and exclude 21 |
| Clinical outcomes of autologous adipose-derived mesenchymal stem cell combined with high tibial osteotomy for knee osteoarthritis are correlated with stem cell stemness and senescence | Interventions other than MSC therapy only | Intervention group: HTO + autologous AD-MSCs injection group Control group: group treated with HTO only High tibial osteotomy (HTO), n=45 |
| Mechanisms of treatment effects using allogeneic, umbilical cord-derived mesenchymal stromal stem cells (MSCs) in knee osteoarthritis: a pharmacological clinical study protocol | Interventions other than MSC therapy only | Can't get the data?! !NCT06078059, Recruitment completed!Fit: Medium Bio:Knee puncture + UCB-MSCs Other:Knee puncture |
| A comparative study between adipocyte-derived mesenchymal stem cell versus intra-articular hyaluronic acid interventions in early osteoarthritis: prospective Randomized Open, Blinded End-point | Interventions other than MSC therapy only | Intervention group: treated with 50 million units of adipose-derived mesenchymal stem cells (ADSCs) in combination with 3 milliliters of high molecular weight hyaluronic acid (HA)Control group: active control group receiving only 3 ml hyaluronic acid (HA) treatmentFit: very high TCTR20230929002 |
| Clinical efficacy of intra-articular mesenchymal stem cells for the treatment of knee osteoarthritis: a double blinded, prospective, randomized, controlled clinical trial | Interventions other than MSC therapy only | Intervention group: high-dose SVF group, low-dose SVF group Control group: placebo group (saline injection group) |
| Adult human mesenchymal stem cells delivered via intra-articular injection to the knee following partial medial meniscectomy: a randomized, double-blind, controlled study | No placebo | Intervention group A: received 50 × 10 allogeneic MSC injections Intervention group B: received 150 × 10 allogeneic MSC injection Control group: sodium hyaluronate injection (vector control) NCT00225095 (exclude 16 after reading the full article) |
| Treatment of Knee Osteoarthritis With Allogeneic Bone Marrow Mesenchymal Stem Cells: A Randomized Controlled Trial | No placebo | Intervention group: allogeneic bone marrow MSCs injection group (40×10*6 cells)Control group: hyaluronic acid active control group (60 mg single injection) |
| Platelet-Rich Plasma and Adipose-Derived Mesenchymal Stem Cells in Association with Arthroscopic Microfracture of Knee Articular Cartilage Defects: A Pilot Randomized Controlled Trial | No placebo | MSC+PRP vs. MSC |
| [CURATIVE EFFECT OF HUMAN UMBILICAL CORD MESENCHYMAL STEM CELLS BY INTRA-ARTICULAR INJECTION FOR DEGENERATIVE KNEE OSTEOARTHRITIS] | No placebo | Dry MSC with HA |
| Injectable cultured bone marrow-derived mesenchymal stem cells in varus knees with cartilage defects undergoing high tibial osteotomy: a prospective, randomized controlled clinical trial with 2 years' follow-up | No placebo | MSC+HA vs. |
| The Effects of Ultrasound-guided Corticosteroid Injection Compared to Mesenchymal Stem Cell Injection in Patients with Grade II and III Knee Osteoarthritis: A Randomized Double-blind Controlled Trial | No placebo | Intervention group: single injection of mesenchymal stem cells (MSCs) Control group: single injection of tretinoinIntervention group: tretinoin + MSCs |
