# Supplementary material for: Contextual effects of mesenchymal stem cell injections for knee osteoarthritis: systematic review and meta-analysis of randomized controlled trials
Source: Front Med (Lausanne). 2025 Sep 17;12:1636181. doi: 10.3389/fmed.2025.1636181 (PMC12487426; doi:10.3389/fmed.2025.1636181)
Supplement: Supplementary file 4 [file Table_4.docx]

# Supplementary File 4: Sensitivity Analyses

## Sensitivity Analysis for Function Outcome at 6 Months





The plot displays the pooled Ratio of Means (ROM) with 95% confidence intervals after omitting one study at a time. The 'Overall pooled estimate' represents the primary meta-analysis result from all studies, included for comparison.

## Sensitivity Analysis for Function Outcome at 12 Months





The plot displays the pooled Ratio of Means (ROM) with 95% confidence intervals after omitting one study at a time. The 'Overall pooled estimate' represents the primary meta-analysis result from all studies, included for comparison.

## Sensitivity Analysis for Pain Outcome at 6 Months





The plot displays the pooled Ratio of Means (ROM) with 95% confidence intervals after omitting one study at a time. The 'Overall pooled estimate' represents the primary meta-analysis result from all studies, included for comparison.

## Sensitivity Analysis for Pain Outcome at 12 Months





The plot displays the pooled Ratio of Means (ROM) with 95% confidence intervals after omitting one study at a time. The 'Overall pooled estimate' represents the primary meta-analysis result from all studies, included for comparison.
